# Conformational Variability Correlation Prediction of Transmissibility and Neutralization Escape Ability for Multiple Mutation SARS-CoV-2 Strains using SSSCPreds

**DOI:** 10.1021/acsomega.1c03055

**Published:** 2021-07-16

**Authors:** Hiroshi Izumi, Laurence A. Nafie, Rina K. Dukor

**Affiliations:** † National Institute of Advanced Industrial Science and Technology (AIST), AIST Tsukuba West, 16-1 Onogawa, Tsukuba, Ibaraki 305-8569, Japan; ‡ Department of Chemistry, Syracuse University, Syracuse, New York 13244-4100, United States; § BioTools Inc., Bee Line Hwy, Jupiter, Florida 33458, United States

## Abstract

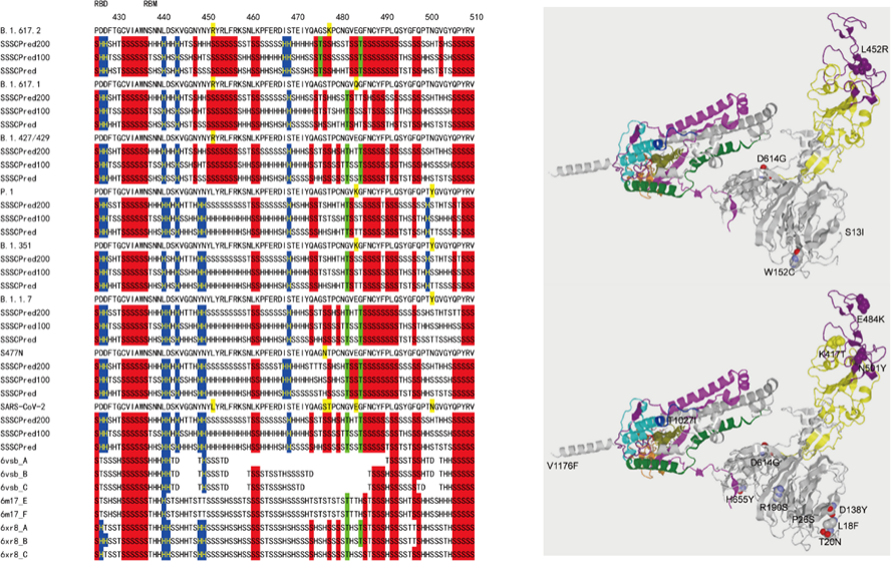

Identifying the fundamental cause of transmissibility of multiple mutation strains and vaccine nullification is difficult in general and is a source of significant concern. The conformational variability of the mutation sites for B.1.617.2 (Δ), B.1.617.1 (κ), B.1.427/429 (ε), P.1 (γ), B.1.351 (β), B.1.1.7 (α), S477N, and the wild-type strain has been assessed using a deep neural-network-based prediction program of conformational flexibility or rigidity in proteins (SSSCPreds). We find that although the conformation of G614 is rigid, which is assigned as a left-handed (LH) α-helix-type one, that of D614 is flexible without the hydrogen bonding latch to T859. The rigidity of glycine, which stabilizes the local conformation more effectively than that of aspartic acid with the latch, thereby contributes to the reduction of S1 shedding, high expression, and increase in infectivity. The finding that the sequence flexibility/rigidity map pattern of B.1.1.7 is similar to that of the wild-type strain but is largely different from those of B.1.351 and P.1 correlates with the minor escape ability of B.1.1.7. The increased rigidity of the amino acid sequence YRYRLFR from the SSSCPreds data of B.1.427/429 near the L452R mutation site contributes to the 2-fold increased B.1.427/B.1.429 viral shedding in vivo and the increase in transmissibility relative to wild-type circulating strains in a similar manner to D614G. The concordance and rigidity ratios of multiple mutation strains such as B.1.617.2 against the wild-type one at the receptor-binding domain (RBD) and receptor-binding motif (RBM) regions provide a good indication of the transmissibility and neutralization escape ability except for binding affinity of mutation sites such as N501Y.

## Introduction

**Figure 1:**
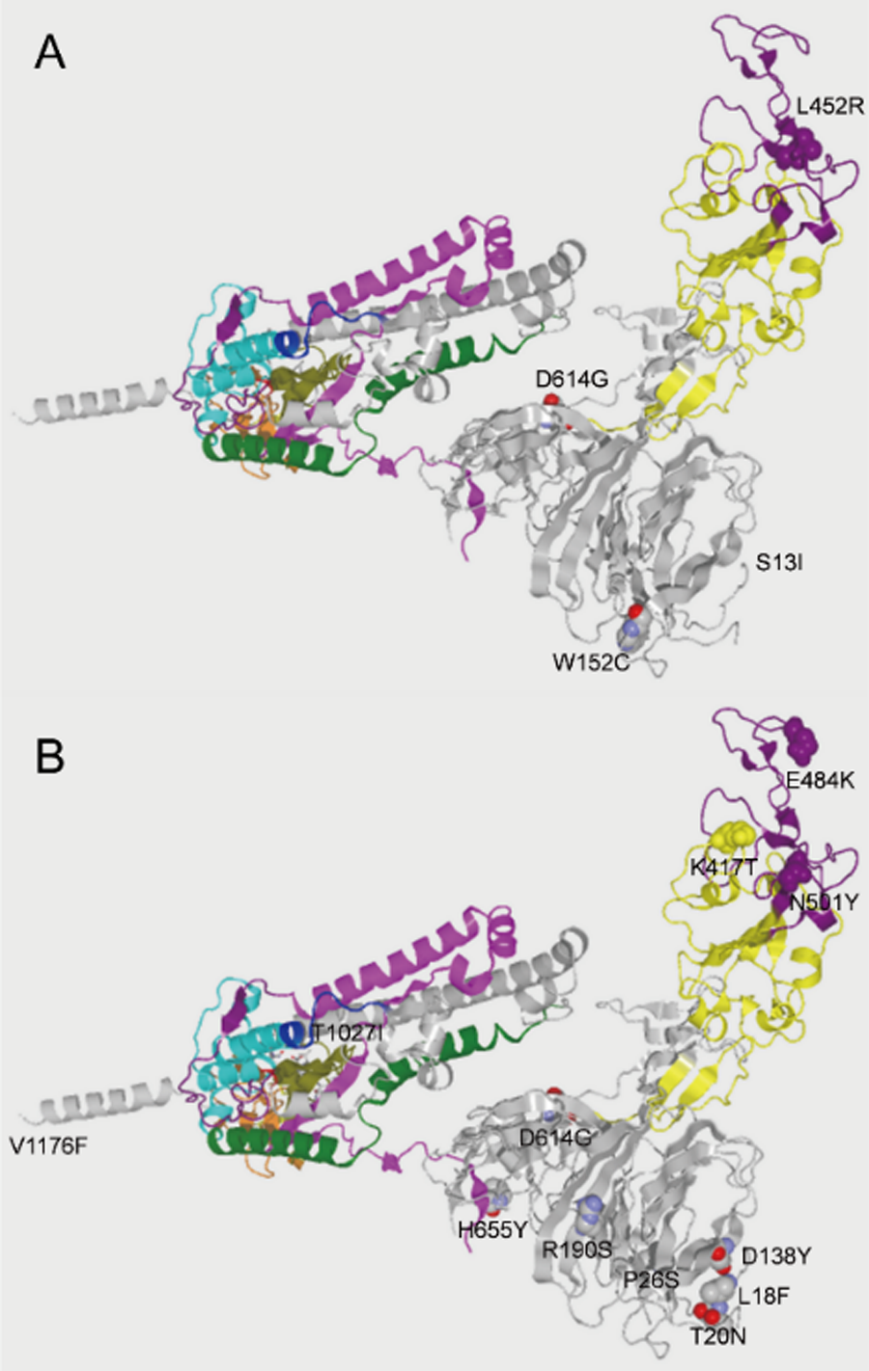
Mutation sites of (A) B.1.427/429 and (B) P.1 on Cryo-EM structures of 6xr8_A (gray: N-terminal domain, subdomain 1, and subdomain 2; yellow: receptor-binding domain; purple: receptor-binding motif; magenta: upstream helix and linker region; cyan: connecting region; green: heptad repeat 1; silver: central helix; olive: β-hairpin; and orange: subdomain 3).

Multiple mutation SARS-CoV-2 strains such as B.1.427/429 (ε)^1^ and P.1 (γ)^2^ have been newly registered as variants of concern by the Centers for Disease Control and Prevention (CDC). In January 2021, novel strains B.1.427/429, which contain S13I, W152C, L452R, and D614G mutations in the spike protein, were found in California (Figure 1A).^1^ The change of transmissibility was largely ascribed to the mutations in receptor-binding domain (RBD). Although B.1.427/429 exhibited an 18.6–24% increase in transmissibility relative to wild-type circulating strains,^1^ a quantitative deep mutational scanning of L452R indicated the constant binding affinity against the wild-type one in contrast with the high expression.^3^ B.1.617, which is classified as a variant of interest by CDC is prevalent in India.^4^ Most recently, B.1.617.2 (Δ) has also been classified as a variant of concern. The main protein substitutions are L452R, E484Q (or T478K), D614G, and P681R.

On the other hand, P.1 that emerged in Brazil has K417T, E484K, N501Y, and D614G mutations (Figure 1B).^2^ One case of SARS-CoV-2 reinfection was associated with the P.1 strain in Manaus.^2^ P.1 was resistant to neutralization by convalescent plasma (3.4-fold) and vaccinee sera (3.8–4.8-fold).^5^ Although the high binding affinity of the mutation site such as N501Y in B.1.1.7 (α)^6^ expanded from UK and in B.1.351 (β)^7^ circulated from South Africa, which was measured by the quantitative deep mutational scanning, can rationalize the increased infections,^3^ identifying the fundamental cause of vaccine nullification is difficult in general, and is a source of significant concern. Furthermore, complementary techniques for the analysis of mutation sites at flexible regions, in which the position of atoms could not be determined by cryo-electron microscopy (Cryo-EM), such as the furin cleavage site of SARS-CoV-2, are needed.

After March 2020, only two mutations, RNA-directed RNA polymerase P323L (ORF1ab P4715L, ORF1b P314L) and spike protein D614G had nearly overwhelmed the original mutation sites (https://nextstrain.org/ncov/global?c=gt-S_614). B.1.1.7 is prevalent all over the world, but the N501Y frequency of phylogeny is about 60–70% (https://nextstrain.org/ncov/global?c=gt-S_501). As for the receptor-binding motif (RBM) of spike protein, before B.1.1.7, B.1.351, and P.1, the mutation of S477N has expanded in Australia and in Europe but has not led to an extension of the pandemic (https://nextstrain.org/ncov/global?c=gt-S_477).

Conformational variability of the mutation site is one of the factors that deeply relate to the infection of SARS-CoV-2.^3,8^ Recently, we reported a deep neural-network-based prediction program of conformational flexibility or rigidity in proteins (SSSCPreds)^8^ using supersecondary structure code (SSSC).^9−11^ The sequence flexibility/rigidity map of SARS-CoV-2 RBD, obtained from SSSCPreds, resembles the sequence-to-phenotype maps of ACE2-binding (angiotensin-converting enzyme 2-binding) affinity and expression, which were experimentally obtained by the deep mutational scanning.^3^ In this paper, we report that the conformational variability assessment using SSSCPreds rationalizes well the transmissibility and the neutralization escape ability of SARS-CoV-2 strains.

## Results and Discussion

### D614G Mutation

As shown above, the D614G variant is now the dominant form worldwide.^12^ Gobeil and co-workers described that Cryo-EM structures reveal altered RBD disposition; antigenicity and proteolysis experiments reveal structural changes and enhanced furin cleavage efficiency of the G614 variant.^12^ However, the underlying factor of why glycine, and not other amino acids, can induce the effective strain replacement has not been explained.

**Figure 2:**
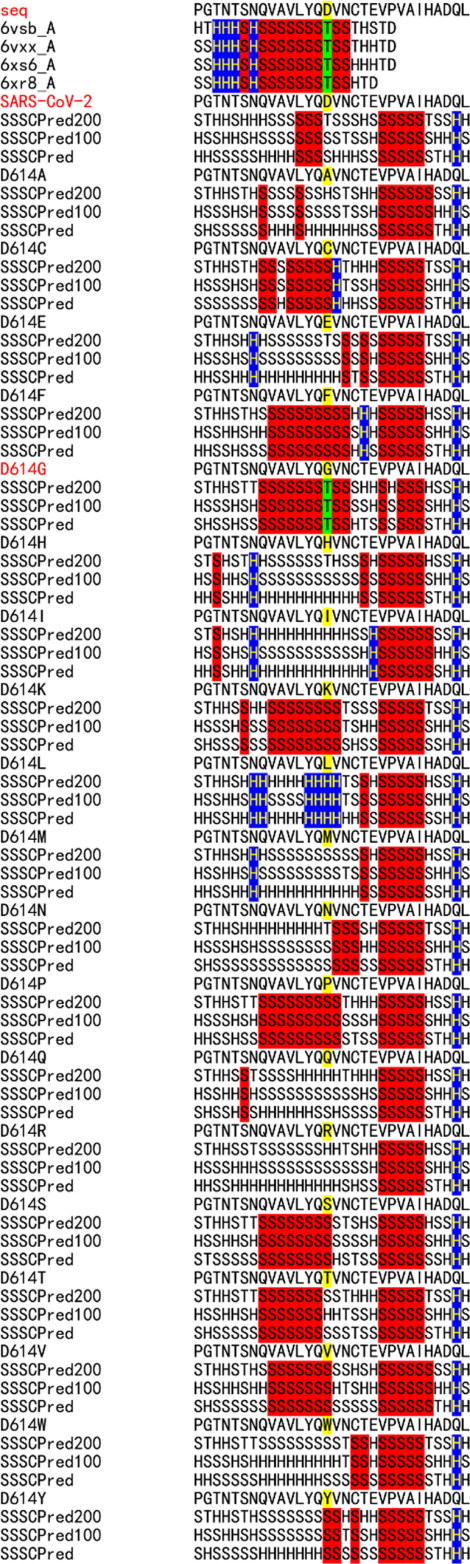
Sequence flexibility/rigidity maps of all of the single amino acid mutations at the D614G mutation site (blue: identical α-helix-type conformations; red: identical β-sheet-type conformations; and green: identical other-type conformations).

**Figure 3:**
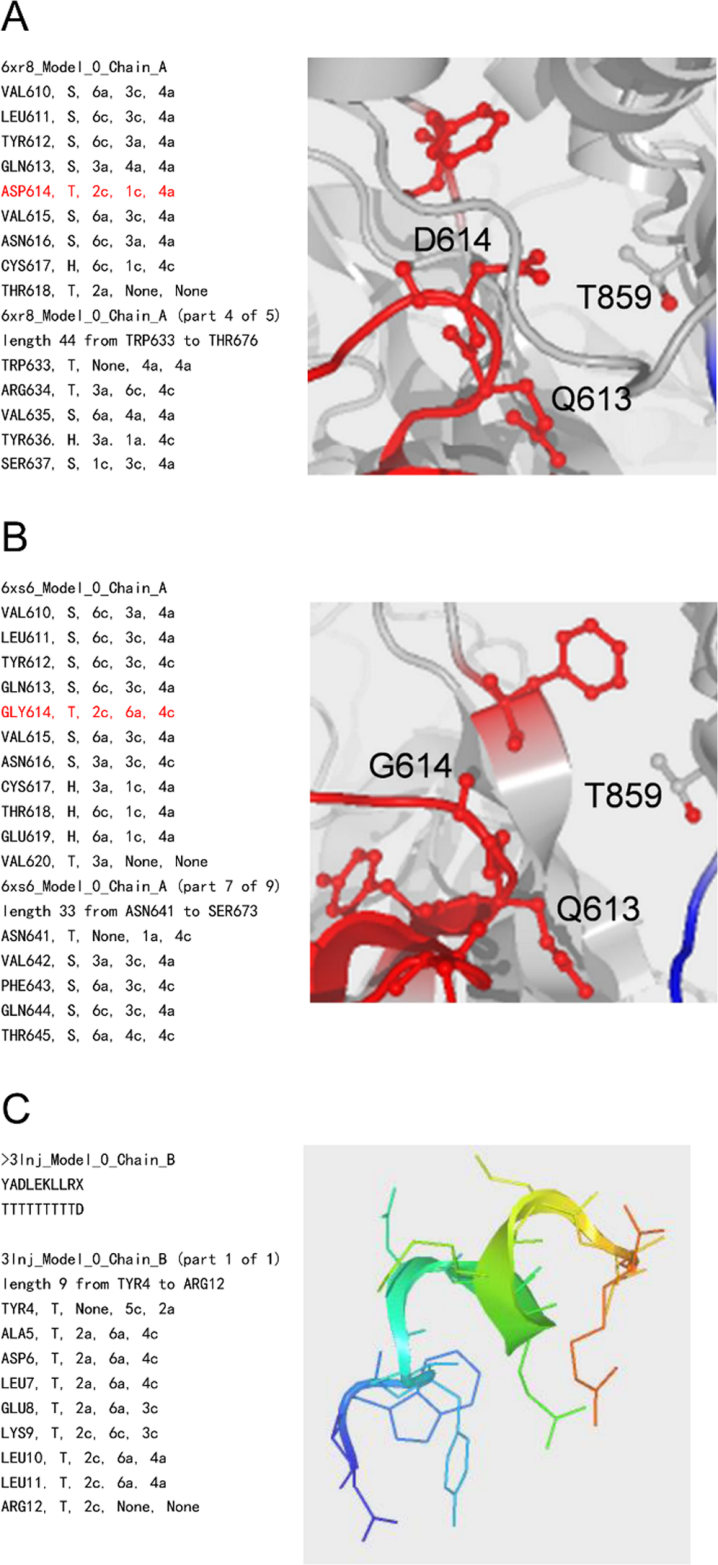
Assignment of conformational codes^8−11^ for (A) D614 of 6xr8_A,^15^ (B) G614 of 6xs6_A,^16^ and (C) D-peptide inhibitor 3lnj_B^17^ with LH α-helix.

The sequence flexibility/rigidity maps of all of the single amino acid mutations at the D614G mutation site using SSSCPreds indicate that only the mutation to glycine makes the other-type conformation (“T” conformation) rigid and reproduces the observed “T” conformations of Cryo-EM structures (Figure 2). On the other hand, although SSSCPred200 suggests the “T” conformation for D614, SSSCPred100 and SSSCPred predict the β-sheet-type conformations (“S” conformations). This means that the site of D614 is flexible without the hydrogen bonding latch between D614 and T859 (Figure 3).

Both observed “T” conformations of Cryo-EM structures for D614 and G614 (6xr8 and 6xs6) are judged as the same conformation, which is assigned as a left-handed (LH) α-helix-type one,^13^ using the SSSCview program with the Protein Data Bank (PDB)^14^ data of 6xr8,^15^ 6xs6,^16^ and 3lnj^17^ (Figure 3). In general, the LH α-helix is stabilized by only glycine because glycine does not have chirality. It is suggested that the rigidity of glycine, which stabilizes the local conformation more effectively than that of aspartic acid with the latch, thereby contributes to the reduction of S1 shedding,^18^ high expression, and increase in infectivity without the latch between D614 and T859.

### B.1.1.7, B.1.351, and P.1

**Figure 4:**
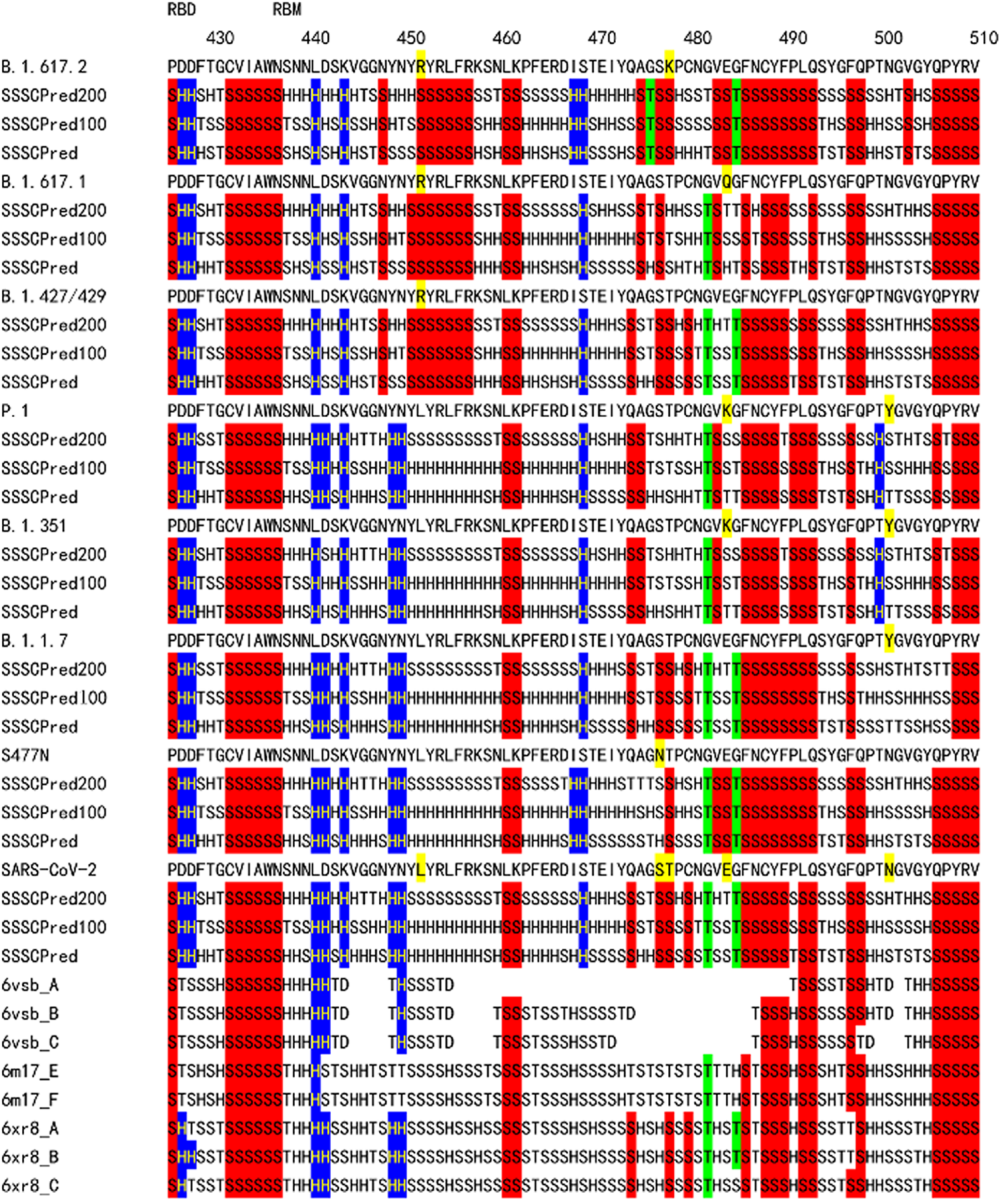
Sequence flexibility/rigidity map of the RBM regions of B.1.617.2, B.1.617.1, B.1.427/429, P.1, B.1.351, B.1.1.7, S477N, and the wild-type strain. The identical SSSC sequences among the predicted ones by three deep neural-network-based systems and the corresponding observed ones are colored (blue: identical α-helix-type conformations; red: identical β-sheet-type conformations; and green: identical other-type conformations). RBM: receptor-binding motif.

As shown in Figure 4, the SSSCPreds data of the expanded S477N variant before B.1.1.7 and B.1.351 indicate that the S477N mutation increases the rigidity of the protein foundation GVEGFNCYFPLQ. The foundation is located on the edge of flexible regions, in which the position of atoms could not be determined. The ratio of frequencies of the S477N mutation has gradually increased, but the mutation has not contributed to the pandemic. The SSSCPreds data of the N501Y mutation for B.1.1.7 show a similar increased stability of the foundation, but the sequence flexibility/rigidity map patterns of B.1.1.7 and the wild-type strain closely resemble one another. This finding correlates with the minor escape ability of B.1.1.7.^19,20^ On the other hand, the sequence flexibility/rigidity map patterns of B.1.351 and P.1 are largely different from that of the wild-type strain. The high ACE2-binding affinity of the single N501Y mutation has been reported.^3^ Although the sequence flexibility/rigidity map cannot predict the high binding affinity of the mutation site such as N501Y, which was measured by the quantitative deep mutational scanning, it can rationalize the neutralization escape ability well.

**Figure 5:**
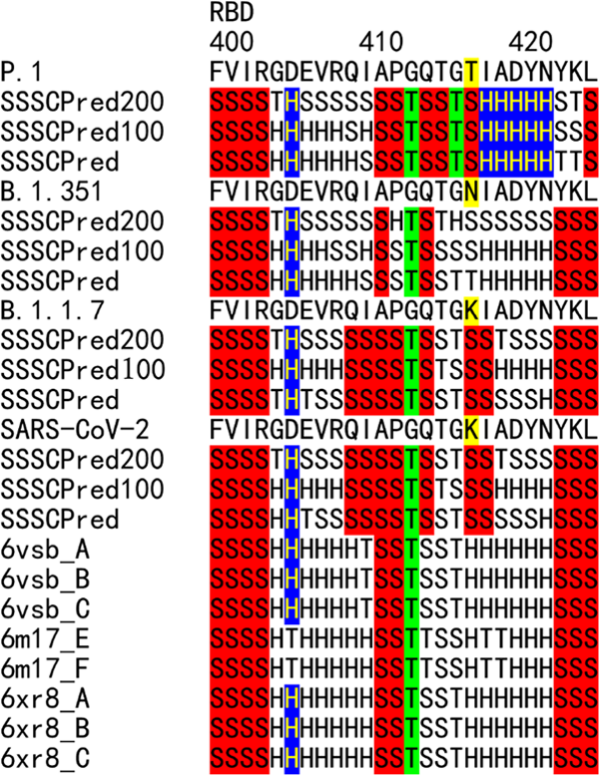
Sequence flexibility/rigidity map of the RBD regions of P.1, B.1.351, B.1.1.7, and the wild-type strain near K417N/T mutation sites. The identical SSSC sequences among the predicted ones by three deep neural-network-based systems and the corresponding observed ones are colored (blue: identical α-helix-type conformations; red: identical β-sheet-type conformations; and green: identical other-type conformations). RBD: receptor-binding domain.

Recently, Dejnirattisai and co-workers reported that P.1 is significantly less resistant to naturally-acquired or vaccine-induced antibody responses than B.1.351, suggesting that changes outside the RBD impact neutralization.^19^ The SSSCPreds data of K417T (P.1) and K417N (B.1.351) indicated the large difference of rigidity between K417N and K417T (Figure 5). K417N with the more flexible SSSC sequence can escape neutralization more effectively than K417T with the less flexible one. It corresponds to the reports that the escape ability of P.1 with K417T is weaker than that of B.1.351 with K417N.^19,20^

### B.1.427/429 and B.1.617.2

The SSSCPreds data of B.1.427/429 and B.1.617.2 near the L452R mutation site are largely different from those of B.1.1.7, B.1.351, P.1, and the wild-type strain (Figure 4). The L452R mutation increases the rigidity of the amino acid sequence YRYRLFR. The sequence is located on the edge of flexible regions (6vsb_A), in which the position of atoms could not be determined. The N501Y mutation site is also located on the opposite edge of flexible regions. It is suggested that the increased rigidity of the edge of flexible regions stabilizes the RBM structure of B.1.427/429 and contributes to the high expression observed by the quantitative deep mutational scanning.^3^ Actually, the other predicted largely-increased rigidity of L452K mutation also corresponds to the observed high expression (Figure S1).^3^ The increased rigidity from the SSSCPreds data of L452R mutation seems to rationalize well the 2-fold increased B.1.427/B.1.429 viral shedding in vivo and the 18.6–24% increase in transmissibility relative to wild-type circulating strains in a similar manner to D614G.^1^ Furthermore, it is suggested that the largely different sequence flexibility/rigidity map patterns of B.1.427/429 and B.1.617.2 from that of the wild-type strain can also rationalize the neutralization escape ability.

**Figure 6:**
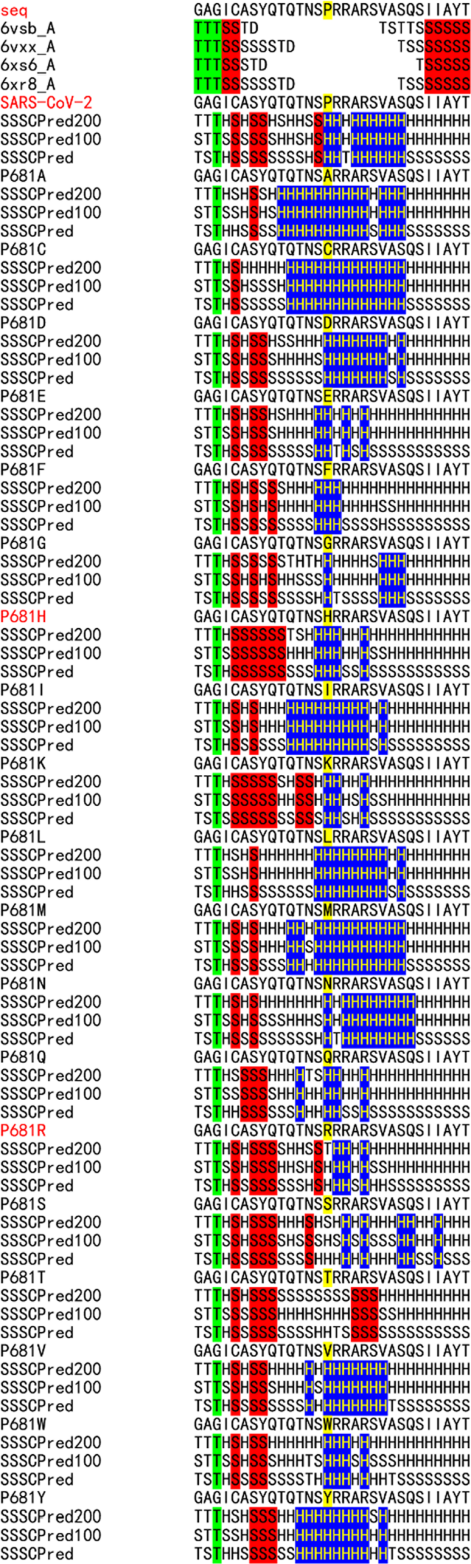
Sequence flexibility/rigidity maps of all of the single amino acid mutations at the P681H/R mutation sites (blue: identical α-helix-type conformations; red: identical β-sheet-type conformations; and green: identical other-type conformations).

B.1.1.7 and B.1.617.2 strains have the P681H/R mutation sites at the furin cleavage site of SARS-CoV-2. The position of atoms at the mutation sites could not be determined by Cryo-EM (Figure 6). The sequence flexibility/rigidity map patterns of the P681H/R mutation sites are more flexible than that of the wild-type strain. It may suggest that the sites are cleaved more easily.

### Quantification

The binding affinity of the mutation site such as N501Y cannot be predicted by SSSCPreds because it is decided by the interaction between two proteins. However, the higher concordance ratio of B.1.1.7 than those of the other multiple strains supports the minor escape ability (Table 1). On the other hand, the rigidity ratios of multiple mutation strains against the wild-type one correlate with those thermodynamical stability and the amounts of expression in comparison with B.1.427/429 and the wild-type strain (Table 1).^1^ Most recently, Wall and co-workers reported that neutralizing antibody titers (NAbTs) were 5·8-fold reduced against B.1.617.2 relative to wild-type (95% CI 5·0–6·9), significantly more reduced than against B.1.1.7 (2·6-fold vs wild-type, 95% CI 2·2–3·1), and on a similar order to the reduction observed against B.1.351 (4·9-fold vs wild-type, 95% CI 4·2–5·7).^21^ The deviation of rigidity ratios from 1.0 also correlates with the neutralization escape ability well. Although a thorough check of the sequence flexibility/rigidity map patterns is necessary, the concordance and rigidity ratios provide a good indication of the transmissibility and neutralization escape ability.

**Table 1:** Concordance and Rigidity Ratios of Multiple Mutation Strains Against the Wild-Type One at the Receptor-Binding Domain (RBD) and Receptor-Binding Motif (RBM) Regions

strains	concordance ratio (RBD)	concordance ratio (RBM)	rigidity ratio (RBD)	rigidity ratio (RBM)
B.1.1.7	0.97	0.94	0.98	0.93
B.1.351	0.92	0.83	0.90	0.85
P.1	0.90	0.85	0.99	0.89
B.1.427/429	0.95	0.85	1.05	1.19
B.1.617.1	0.91	0.74	1.01	1.04
B.1.617.2	0.89	0.72	1.10	1.30

## Conclusions

In conclusion, the conformational variability of the mutation sites for B.1.617.2, B.1.617.1, B.1.427/429, P.1, B.1.351, B.1.1.7, S477N, and the wild-type strain has been assessed using SSSCPreds. The SSSCPreds data of the D614G mutation suggest that although the G614-induced conformation is stabilized in the same way as found in a LH α-helix, the D614 conformation is flexible without the hydrogen bonding latch between D614 and T859. The stability of the G614 conformation, without a latch possibility, seems to correlate with the reduction of S1 shedding, high expression, and increased infectivity.

The finding that the sequence flexibility/rigidity map patterns of B.1.1.7 and the wild-type strain have an extreme resemblance and correlates with the minor escape ability of B.1.1.7 well. K417N with the more flexible SSSC sequence from the SSSCPreds data of K417T (P.1) and K417N (B.1.351) can escape neutralization more effectively. The increased rigidity of the amino acid sequence YRYRLFR for the L452R mutation stabilizes the RBM structure of B.1.427/429 and contributes to the high expression observed by the quantitative deep mutational scanning. The prediction data of mutation sites using SSSCPreds with the quantification data is helpful for the interpretation of transmissibility and neutralization escape ability for multiple mutation strains.

## Computational Methods

The FASTA-format files containing the original and mutation sequences of protein subunits were converted to the predicted SSSCs using the SSSCPreds program (available online at https://staff.aist.go.jp/izumi.h/SSSCPreds/index-e.html).^8^ The SSSCPreds program can be used easily by the window-menu operation. The FASTA-format files containing the amino acid sequences and SSSCs of protein subunits were obtained from the observed PDB files^14^ using the SSSCview program (available online at https://staff.aist.go.jp/izumi.h/SSSCPreds/index-e.html).^11^

The concordance ratios of multiple mutation strains against the wild-type one at the RBD and RBM regions were calculated using agreements of identical α-helix-type conformations, identical β-sheet-type conformations, identical other-type conformations, and flexible conformations. The rigidity ratios of multiple mutation strains against the wild-type one at the RBD and RBM regions were obtained using total counts of numbers of identical α-helix-type conformations, identical β-sheet-type conformations, and identical other-type conformations.

## Supporting Information Available

Sequence flexibility/rigidity maps of all of the single amino acid mutations at L452R mutation site of B.1.427/429 and comparison of SSSCPreds data for B.1.617.2, B.1.617.1, B.1.427/429, P.1, B.1.351, B.1.1.7, and original sequences of SARS-CoV-2 with observed PDB data (PDF)
