# Supplementary material for: Conformational Variability Correlation Prediction of Transmissibility and Neutralization Escape Ability for Multiple Mutation SARS-CoV-2 Strains using SSSCPreds
Source: ACS Omega. 2021 Jul 16;6(29):19323–9. doi: 10.1021/acsomega.1c03055 (PMC8320097; doi:10.1021/acsomega.1c03055)
Supplement: Supplementary file 1 — ao1c03055_si_001.pdf [file ao1c03055_si_001.pdf]

Supporting Information for

Conformational Variability Correlation Prediction of Transmissibility and Neutralization Escape Ability for Multiple Mutation SARS-CoV-2 Strains using SSSCPreds

Hiroshi Izumi,[*,†] *Laurence A. Nafie,*[‡,§] *and Rina K. Dukor*[§]

[†]National Institute of Advanced Industrial Science and Technology (AIST), AIST Tsukuba West, 16-1 Onogawa, Tsukuba, Ibaraki 305-8569, Japan
[‡]Department of Chemistry, Syracuse University, Syracuse, New York 13244-4100, United States
[§]BioTools Inc., Bee Line Hwy, Jupiter, Florida 33458, United States

e-mail: izumi.h@aist.go.jp

Table of contents

Figure S1	S2
Figure S2	S3-S17

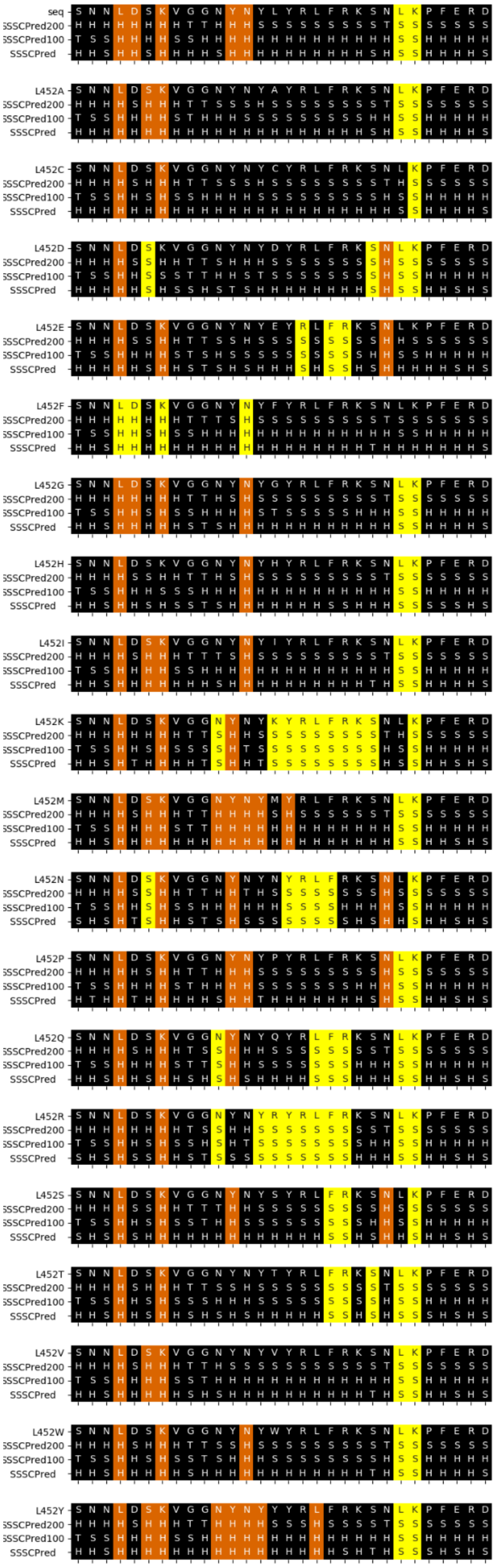

Figure S1. Sequence flexibility/rigidity maps of all of the single amino acid mutations at L452R mutation site of B.1.427/429 (orange: identical α-helix-type conformations; yellow: identical β-sheet-type conformations; purple: identical other-type conformations; black: flexible conformations).

Figure S2. Comparison of SSSCPreds data for B.1.617.2, B.1.617.1, B.1.427/429, P.1, B.1.351, B.1.1.7, and original sequences of SARS-CoV-2 with observed PDB data.

		NTD (1 … 10 … 20 … 30 … 40 … 50 … 60 … 70 … 80)	
B. 1. 617. 2		MFVFLVLLPLVSSQCVN—LRTRTQLPPAYTN--SFTRGVYYPDKVFRSSVLHSTQDLFLPFFSNVTWFHAIHVSGTNGTKRFDNPVLP	
SSSCPred200		HHHHHHHHHHHHHHHHH—HHHSSSSSSSSSS--SSSSTSSSSSSSSHHHHHHHHHHHHHHHTHHSSHHHHHHHHHHHHTSSSSSSSSSS	
SSSCPred100		HHHHHHHHHHHHHHHSSS—HHHHHTSSSSSSS--SSSSSSSSHHHSSSSSSSSSSHSSHHHHHTHHSHSSSSSSSSSSHHTSSSSHSSSSS	
SSSCPred		HHHHHHHHHHHHSSSSS—SSSHSHSSHSSSS--HSSSSSSSSSSSSSSSHSSHSHHHHHHHHHHHHSHSSSSSSHSSHTTSSSSHTHSSS	
B. 1. 617. 1		MFVFLVLLPLVSSQCVN—LTTRTQLPPAYTN—SFTRGVYYPDKVFRSSVLHSTQDLFLPFFSNVTWFHAIHVSGTNGTKRFDNPVLP	
SSSCPred200		HHHHHHHHHHHHSHHHH—SSSSSSSSSSSSS—SSSSTSSSSSSSSHHHHHHHHHHHHHHHTHHSSHHHHHHHHHHHHTSSSSSSSSSS	
SSSCPred100		HHHHHHHHHHHHHSSSS—HHHHSTSSSSSSS—SSSSSSSSHHHSSSSSSSSSSHSSHHHHHTHHSHSSSSSSSSSSHHTSSSSHSSSSS	
SSSCPred		HHHHHHHHHHSHSSSSS—SSSHSSSSSSSSS—TSSSSSSSSSSSSSSSHSSHSHHHHHHHHHHHHSHSSSSSSHSSHTTSSSSHTHSSS	
B. 1. 427/429		MFVFLVLLPLVSIQCVN—LTTRTQLPPAYTN--SFTRGVYYPDKVFRSSVLHSTQDLFLPFFSNVTWFHAIHVSGTNGTKRFDNPVLP	
SSSCPred200		HHHHHHHHHHHHHHHHH—HHSSSSSSSSSSS--SSSSTSSSSSSSSHHHHHHHHHHHHHHHTHHSSHHHHHHHHHHHHTSSSSSSSSSS	
SSSCPred100		HHHHHHHHHHHHHHHSSH—HHHHSTSSSSSSS--SSSSSSSSHHHSSSSSSSSSSHSSHHHHHTHHSHSSSSSSSSSSHHTSSSSHSSSSS	
SSSCPred		HHSSHHHHHHHHHSSSSS—HHSHSSSSSTSSS--TSSSSSSSSSSSSSSSHSSHSHHHHHHHHHHHHSHSSSSSSHSSHTTSSSSHTHSSS	
P. 1		MFVFLVLLPLVSSQCVN—FTNRTQLPSAYTN--SFTRGVYYPDKVFRSSVLHSTQDLFLPFFSNVTWFHAIHVSGTNGTKRFDNPVLP	
SSSCPred200		HHHHHHHHHHHHHHHHSS—SHSSSSSSSHSSS--SSSSTSSSSSSSSHHHHHHHHHHHHHHHTHHSSHHHHHHHHHHHHTSSSSSSSSSS	
SSSCPred100		HHHHHHHHHHHHHSSHHH—HHHHHTSSSHHSS--SSSSSSSSHHHSSSSSSSSSSHSSHHHHHTHHSHSSSSSSSSSSHHTSSSSHSSSSS	
SSSCPred		HHHHHHHHHHSHSSSSS—SSSHSSSSHSSSS--TSSSSSSSSSSSSSSSHSSHSHHHHHHHHHHHHSHSSSSSSHSSHTTSSSSHTHSSS	
B. 1. 351		MFVFLVLLPLVSSQCVN—FTTRTQLPPAYTN--SFTRGVYYPDKVFRSSVLHSTQDLFLPFFSNVTWFHAIHVSGTNGTKRFANPVLP	
SSSCPred200		HHHHHHHHHHHHSHSSS—SSSSSSSSSSSSS--SSSSTSSSSSSSSHHHHHHHHHHHHHHHTHHSSSSSSSSSSSSHSTSSSSSTSSSS	
SSSCPred100		HHHHHHHHHHHHHSSSSS—HHSHSTSSSSSSS--SSSSSSSSHHHSSSSSSSSSSHSSHHHHHTHHSHSSSSSSSSSSHHHSSSSSSSSSS	
SSSCPred		HHHHHHHHHHSHSSSSS—SHSSSSSSSHSSS--TSSSSSSSSSSSSSSSHSSHSHHHHHHHHHHHHSSSSSSSSSSTHHTSSSSSTSSSH	
B. 1. 1. 7		MFVFLVLLPLVSSQCVN—LTTRTQLPPAYTN—SFTRGVYYPDKVFRSSVLHSTQDLFLPFFSNVTWFHAI—SGTNGTKRFDNPVLP	
SSSCPred200		HHHHHHHHHHHHSHHHH—SSSSSSSSSSSSS—SSSSTSSSSSSSSHHHHHHHHHHHSSHHTHHSSSSSSS—SHHHTSSHSSSHSSS	
SSSCPred100		HHHHHHHHHHHHHSSSS—HHHHSTSSSSSSS—SSSSSSSSHHHSSSSSSSSSSHSSHHHHHTHHSSSSSSS—SSHHTSSSSHSSSSS	
SSSCPred		HHHHHHHHHHSHSSSSS—SSSHSSSSSSSSS—SSSSSSSSSSSSSSSSHSSHSHHHHHHHHSSSHSHSSSHH—HTHTTHSSSHTHSSS	
SARS-CoV-2	1	MFVFLVLLPLVSSQCVN—LTTRTQLPPAYTN--SFTRGVYYPDKVFRSSVLHSTQDLFLPFFSNVTWFHAIHVSGTNGTKRFDNPVLP	85
SSSCPred200		HHHHHHHHHHHHSHHHH—SSSSSSSSSSSSS--SSSSTSSSSSSSSHHHHHHHHHHHHHHHTHHSSHHHHHHHHHHHHTSSSSSSSSSS	
SSSCPred100		HHHHHHHHHHHHHSSSS—HHHHSTSSSSSSS--SSSSSSSSHHHSSSSSSSSSSHSSHHHHHTHHSHSSSSSSSSSSHHTSSSSHSSSSS	
SSSCPred		HHHHHHHHHHSHSSSSS—SSSHSSSSSSSSS--TSSSSSSSSSSSSSSSHSSHSHHHHHHHHHHHHSHSSSSSSHSSHTTSSSSHTHSSS	
6vsb_A		TSSS—THHSTSSSHSHSSSSTSSSSSSSSSSSSTSSSSSSTD TSSSSSS	
6vsb_B		TSSS—SHHSTSSSHSHSSSSTSSSSSSSTSSSSTSSSSSSTD TSSSS	
6vsb_C		TSSS—THHSTSSSHSHSSSSTSTSSSSSTSSSSTSSSSSSTD TSSSSS	
6vxx_A		TSSS—HTHSTSSSHSHSSSSTSSSSSSSSSSSSTSSSSSSSTSTD TTSSSS	
6vxx_B		TSSS—HTHSTSSSHSHSSSSTSSSSSSSSSSSSTSSSSSSSTSTD TTSSSS	
6vxx_C		TSSS—HTHSTSSSHSHSSSSTSSSSSSSSSSSSTSSSSSSSTSTD TTSSSS	
6vyb_A		TSSS—HTHSTSSSHSTSSSSTSSSSSSSSSSSSTSSSSSSSTSTD TSSS	
6vyb_B		TSSS—HTHSTSSSHSTSSSSTSSSSSSSSSSSSTSSSSSSTD TSSSS	
6vyb_C		TSSS—HTHSTSSSHSTSSSSTSSSSSSSSSSSSTSSSSSSTD TSSSS	
6xs6_A		TSSS—SHHSTSSSHSTSSSSTSSSSSSSSSSSSTSSSSSSSHSTD TTSSSS	
6xs6_B		TSSS—SHHSTSSSHSTSSSSTSSSSSSSSSSSSTSSSSSSSHSTD TTSSSS	
6xs6_C		TSSS—SHHSTSSSHSHSSSSTSSSSSSSSSSSSTSSSSSSSHSTD TTSSSS	
6xr8_A		TSSS—THSSSSSSSSSSS—THTSTSSSHSHSSSSTSSSSSSSSSSSSTSSSSSSSSSTD TSSSTSSSS	
6xr8_B		TSSS—THSSSSSSSSSSS—SHTSTSSSHSHSSSSTSSSSSSSSSSSSTSSSSSSSSSTD TSSSTSSSS	
6xr8_C		TSSS—SHSSSSSSSSSSS—THTSTSSSHSHSSSSTSSSSSSSSSSSSTSSSSSSSSSTD TSTSTSSSS	
6xra_A			
6xra_B			
6xra_C			

```
                         NTD
                             90        100       110       120       130       140       150       160       170
B.1.617.2                FNDGVYFASTEKSNIIRGWIFGTTLDSKTQSLLIVNNATNVVIKVCEFQFCNDPFLDVYYHKNNKSWMESE--VYSSANNCTFEY
SSSCPred200              SSHTSSSSSSHHHHHHSSSSHSSSTSSSSSSSSSSTTSSSSSSSHSHHHSTSHHHSHSHSSTTSSSSSSS--SSSHSHTSSSHS
SSSCPred100              SSHTHSSHHHHHHHHHHHHSSSTSSHHTSSSSSSSSSSHHSSSSSSSHSSHSHSHHHHHHHHHHHHHSHHHHH--HHHHHHTSSSSS
SSSCPred                 STSTSSSSSSHHSTSSSSSSSSSSSSHHHSSSSSSSSSHHSSSHSSSSSSSSSSSHHHHHHHSSSHTHHHHSH--HHHHSHHHSSSS
B.1.617.1                FNDGVYFASTEKSNIIRGWIFGTTLDSKTQSLLIVNNATNVVIKVCEFQFCNDPFLDVYYHKNNKSWMKSEFRVYSSANNCTFEY
SSSCPred200              SSHTSSSSSSHHHHHHSSSSHSSSTSSSSSSSSSSTTSSSSSSSHSHHHSTSSSHSSSSSSTTSSHSSSSSSSSSSHHTSSSSS
SSSCPred100              SSHTHSSHHHHHHHHHHHHSSSTSSHHTSSSSSSSSSSHHSSSSSSSHSSHSHSHHHHHHHHHHHSHHHHTSSSSSSSTTSTSSSSS
SSSCPred                 STSTSSSSSSHHSTSSSSSSSSSSSSHHHSSSSSSSSSSHSSSHSSSSSSSSSSSHHHHHHSSSHHTSSSSHHSSSSSSSTSHHSSSS
B.1.427/429              FNDGVYFASTEKSNIIRGWIFGTTLDSKTQSLLIVNNATNVVIKVCEFQFCNDPFLGVYYHKNNKSCMESEFRVYSSANNCTFEY
SSSCPred200              SSHTSSSSSSHHHHHHSSSSSSSSTSSSSSSSSSSTTSSSSSSSHTHHHSHSSSSSSSSSSSTTSSSSSSSSSSSSSHHTSSSSS
SSSCPred100              SSHTHSSHHHHHHHHHHHHSHSTSSHHTSSSSSSSSSSHHSSSSSSSHSSHSHSHTSTSSSSSTSSSSSSHSSSSSSSTTSTSSSSS
SSSCPred                 STSTSSSSSSHHSTSSSSSSSSSSSSHHHSSSSSSSSSSHSSSHSSSSSSSSSSSSSSSTSSSSHHHHSSSSSSSSSSSSHTSTHSSSS
P.1                      FNDGVYFASTEKSNIIRGWIFGTTLDSKTQSLLIVNNATNVVIKVCEFQFCNYPFLGVYYHKNNKSWMESEFRVYSSANNCTFEY
SSSCPred200              SSHTSSSSSSHHHHHHSSSSSSSSTSSSSSSSSSSTTSSSSSSSHTSHHTHSSSHSSSSSSTTSSSSSSSSSSSSSSSHSTSSSSS
SSSCPred100              SSHTHSSHHHHHHHHHHHHSSSTSSHHTSSSSSSSSSSHHSSSSSSSSSSSSHSHSSTSSSSHHHHSHSSSSSSSSSSTTTTSSSSS
SSSCPred                 STSTSSSSSSHHSTSSSSSSSSSSSSHHHSSSSSSSSSHHSSSHSSSSSHHHHSSSSSSSSSSHHHTSSSSSSSSSSSSHTSSHSSSS
B.1.351                  FNDGVYFASTEKSNIIRGWIFGTTLDSKTQSLLIVNNATNVVIKVCEFQFCNDPFLGVYYHKNNKSWMESEFRVYSSANNCTFEY
SSSCPred200              SSHTSSSSSSSHHHHHHSSSSSSSSTSSSSSSSSSSTTSSSSSSSHTHHHSHSSSHTSSSSSTTSSSSSSSSSSSSSSSHSTSSSSS
SSSCPred100              HHHTHSSHHHHHHHHHHHHSSSTSSHHTSSSSSSSSSSHHSSSSSSSHSSHSHSHTSTSSSSSHHHSHSSSSSSSSSTTSTSSSSS
SSSCPred                 STSTSSSSSSHHSTSSSSSSSSSSSSHHHSSSSSSSSSHHSSSHSSSSSSSSSSSSHHSTSSSSHHHTSSSSSHSSSSSHTSSHSSSS
B.1.1.7                  FNDGVYFASTEKSNIIRGWIFGTTLDSKTQSLLIVNNATNVVIKVCEFQFCNDPFLGV-YHKNNKSWMESEFRVYSSANNCTFEY
SSSCPred200              SSHTSSSSSSSHHHHHHSSSSHSSSSSSSSSSSSSSSTTSSSSSSSHTHHHSHSSSHHS-SSSTSHHSSHSSSSSSSSHSTSSSSS
SSSCPred100              SSHTHSSHHHHHHHHHHHHSSHTSSHHTSSSSSSSSSSHHSSSSSSSHSSHSHSHSSSS-SSHTHHSHSSSSSSSSSTTTTSSSSS
SSSCPred                 STSTSSSSSSHHSTSSSSSSSSSSSSHHHSSSSSSSSSSHSSSHSSSSHSSSSSHHSTS-SSHHHTSSSSSHSSSSSHTSSHSSSS
SARS-CoV-2          86   FNDGVYFASTEKSNIIRGWIFGTTLDSKTQSLLIVNNATNVVIKVCEFQFCNDPFLGVYYHKNNKSWMESEFRVYSSANNCTFEY   170
SSSCPred200              SSHTSSSSSSSHHHHHHSSSSSSSSTSSSSSSSSSSTTSSSSSSSHTHHHSHSSSHTSSSSSTTSSSSSSSSSSSSSSSHSTSSSSS
SSSCPred100              SSHTHSSHHHHHHHHHHHHSHSTSSHHTSSSSSSSSSSHHSSSSSSSHSSHSHSHTSTSSSSSHHHSHSSSSSSSSSTTSTSSSSS
SSSCPred                 STSTSSSSSSHHSTSSSSSSSSSSSSHHHSSSSSSSSSHHSSSHSSSSSSSSSSSSHHSTSSSSHHHTSSSSSHSSSSSHTSSHSSSS
6vsb_A                   HTTTSSSSSTD  THSHSSSSTHHTSHHSSSSTSSSTTHSSSSSHSTSSSSHTSSTTD            TSHHSHSTSTSHSSS
6vsb_B                   HTTTSSSSSHSTSHHSHTSSSTHHTSHHSSSSTSSSHTHSSSSSHSTSSSSHTSSTD            TSSTHTHSTSTSHSSS
6vsb_C                   HTTTSSSSSTD  THSSSSSSSHHTSHHSSSSTTSSHTHSSSSSHSTSSSSHSSTD              THHSHSTSTSHSSS
6vxx_A                   HTHTSSSSSSSHHTHSHSSSSTHHTTTHSSSSSSSSSTHSSSSSTSTSSSSHSSSSSTD                      TSHSSS
6vxx_B                   HTHTSSSSSSSHHTHSHSSSSTHHTTTHSSSSSSSSSTHSSSSSTSTSSSSHSSSSSTD                      TSHSSS
6vxx_C                   HTHTSSSSSSSHHTHSHSSSSTHHTTTHSSSSSSSSSTHSSSSSTSTSSSSHSSSSSTD                      TSHSSS
6vyb_A                   HTHTSSSSSSSHHTHSHSSSSTHHTSTTD TSSSSTSTHSSSSSTSTSSSSHTSSSSTD                       THSSS
6vyb_B                   HTHTSSSSSSSHHTHSHSSSSTHHTTTTSSSSSSSSSTHSSSSSTSTSSSSHTSTD                        TTSHSSS
6vyb_C                   HTHTSSSSSSSHHTHSHSSSSTHHTTTHSSSSSSSSSTHSSSSSTSTSSSSHTSSSSTD                      TSHSSS
6xs6_A                   SHHTSSSSSSSHHTHSHSSSSTHHTHTHSSSSSSSSTTHSSSSSHSSSSSSHTSSSSTD                      TSHSSS
6xs6_B                   THHTSSSSSSSHHTHSHSSSSTHHTHTHSSSSSSSSTTHSSSSSHSSSSSSHTSSSSTD                      TSHSSS
6xs6_C                   THHTSSSSSSSHHTHSHSSSSTHHTHTHSSSSSSSSTTHSSSSSHSSSSSSHTSSSSTD                      TSHSSS
6xr8_A                   SSTTSSSSSSSSTTHSHTSSSTHHTHTHSSSSSSSSSHHSSSSSSSTSSSSHSSSSSSSSTTTHTSSSSHSSTHSHSTHSSHSSS
6xr8_B                   SSTTSSSSSSSSTTHSTTSSSTHHTHTHSSSSSSSTSHHSSSSSSSTSSSSHTSSSSSSSTTTHTSSSSHSSTHSHSSHSSHSSS
6xr8_C                   SSTTSSSSSSSSTTHSHTSSSTHHTHTHSSSSSSSTSHHSSSSSSSTSSSSHTSSSSSSSTTTHTSSSSHSTTHSHSSHSSHSSS
6xra_A
6xra_B
6xra_C
```

```
                          NTD
                          171      180       190       200       210       220       230       240       250
B.1.617.2                 VSQPFLMDLEGKQGNFKNLREFVFKNIDGYFKIYSKHTPINLVRDLPQGFSALEPLVDLPIGINITRFQTLLALHRSYLTPGDSS
SSSCPred200               SSSSHHSSSSHHSTTTHHSSSSSSHSHHHHHHHHSHHTHSSSSHSTSSTHHHHHHHSHSSHHSHHHHHHHHHHHHHHHHHSSTHHS
SSSCPred100               SSSHHHSSHSTSSTHTHTSSSSSSHHSSSSSSSSSSTSSHHHSHHSSSTSSHSSSSSSSSSTHTSHHHHHHHHHHHHHHHSHHHSH
SSSCPred                  SSSSSSSHHHTSHTSHHHHHHHHHHHHHHHHHHSSSSHHSSSSSSSTSSSTSHHHHHHHHHSSTSSSHHHHHHHHHHHHHHHHHHHHHH
B.1.617.1                 VSQPFLMDLEGKQGNFKNLREFVFKNIDGYFKIYSKHTPINLVRDLPQGFSALEPLVDLPIGINITRFQTLLALHRSYLTPGDSS
SSSCPred200               SSSSHHSSSSHHSTTTSHSSSSSSHSHHHHHHHHSHHTHSSSSHSTSSTHHHHHHHSHSSSHSHHHHHHHHHHHHHHHHHSSTHHS
SSSCPred100               SSSHHHSSHSTSSTHTHTSSSSSSHHSSSSSSSSSSTSSHHHSHHSSSTSSHSSSSSSSSSTHTSHHHHHHHHHHHHHHHSHHHSH
SSSCPred                  SSSSHHHHHHTSHTSHHHHHHHHHHHHHHHHHHSSSSHHSSSSSSSTSSSTSHHHHHHHHHSSTSSSHHHHHHHHHHHHHHHHHHHHHH
B.1.427/429               VSQPFLMDLEGKQGNFKNLREFVFKNIDGYFKIYSKHTPINLVRDLPQGFSALEPLVDLPIGINITRFQTLLALHRSYLTPGDSS
SSSCPred200               SSSSHHSSSSHHSTTTSHSSSSSSHSHHHHHHHHSHHTHSSSSHSTSSTHHHHHHHSHSSSHSHHHHHHHHHHHHHHHHHSSTHHS
SSSCPred100               SSSHHHSSHSTSSTHTHTSSSSSSHHSSSSSSSSSSTSSHHHSHHSSSTSSHSSSSSSSSSTHTSHHHHHHHHHHHHHHHSHHHSH
SSSCPred                  SSSSHHHHHHTSHTSHHHHHHHHHHHHHHHHHHSSSSHHSSSSSSSTSSSTSHHHHHHHHHSSTSSSHHHHHHHHHHHHHHHHHHHHHH
P.1                       VSQPFLMDLEGKQGNFKNLSEFVFKNIDGYFKIYSKHTPINLVRDLPQGFSALEPLVDLPIGINITRFQTLLALHRSYLTPGDSS
SSSCPred200               SSSSHHSSHSHHSTHTSHHHHHHHHSHHHHHHHHSHHTHSSSSHSTSSTHHHHHHHSHSSSHSHHHHHHHHHHHHHHHHHSSTHHS
SSSCPred100               SSSHHHSSHSTSSTHHHTHSSHSSHHSSSSSSSSSSTSSHHHSHHSSSTSSHSSSSSSSSSTHTSHHHHHHHHHHHHHHHSHHHSH
SSSCPred                  SSSSHHHHHHTSSTSSHSSSSSSSSHHHHHHHHHHSHHSSSSSSSTSSSTSHHHHHHHHHSSTSSSHHHHHHHHHHHHHHHHHHHHHH
B.1.351                   VSQPFLMDLEGKQGNFKNLREFVFKNIDGYFKIYSKHTPINLVRGLPQGFSALEPLVDLPIGINITRFQTLLALHISYLTPGDSS
SSSCPred200               SSSSHHSSSSHHSTTTSHSSSSSSHSHHHHHHHHSHHSHSSSSSSSSSTHHHHHHHSHSSHHHHHHHHHHHHHHHHHHHHHHSSSSHS
SSSCPred100               SSSHHHSSHSTSSTHTHTSSSSSSHHSSSSSSSSSSTSSSSHHHTSSSTSHHSSSSSSSSSTHTSHSHHSSSSSHHHTSSHHHSH
SSSCPred                  SSSSHHHHHHTSHTSHHHHHHHHHHHHHHHHHSSSSSHHSHHSSSSTSSSTSHHHHHHHHHSSTSSSHHHHHHHHHHHHHHHHHHHTHHH
B.1.1.7                   VSQPFLMDLEGKQGNFKNLREFVFKNIDGYFKIYSKHTPINLVRDLPQGFSALEPLVDLPIGINITRFQTLLALHRSYLTPGDSS
SSSCPred200               SSSSHHSSSSHHSTTHSHSSSSSSHSHHHHHHHHSHHTHSSSSHSTSSTHHHHHHHSHSSHHSHHHHHHHHHHHHHHHHHSSTHHH
SSSCPred100               SSSHHHSSHSTSSTHTHTSSSSSSHHSSSSSSSSSSTSSHHHSHHSSSTSSHSSSSSSSSSTHTSHHHHHHHHHHHHHHHSHHHSH
SSSCPred                  SSSSHHHHHHTSSTSHHHHHHHHHHHHHHHHHSSSSSHHSSSSSSSTSSSTSHHHHHHHHHSSTSSSHHHHHHHHHHHHHHHHHHHHHH
SARS-CoV-2          171   VSQPFLMDLEGKQGNFKNLREFVFKNIDGYFKIYSKHTPINLVRDLPQGFSALEPLVDLPIGINITRFQTLLALHRSYLTPGDSS     255
SSSCPred200               SSSSHHSSSSHHSTTTSHSSSSSSHSHHHHHHHHSHHTHSSSSHSTSSTHHHHHHHSHSSSHSHHHHHHHHHHHHHHHHHSSTHHS
SSSCPred100               SSSHHHSSHSTSSTHTHTSSSSSSHHSSSSSSSSSSTSSHHHSHHSSSTSSHSSSSSSSSSTHTSHHHHHHHHHHHHHHHSHHHSH
SSSCPred                  SSSSHHHHHHTSHTSHHHHHHHHHHHHHHHHHHSSSSHHSSSSSSSTSSSTSHHHHHHHHHSSTSSSHHHHHHHHHHHHHHHHHHHHHH
6vsb_A                    SSSSHTD         TSSSSSSSSSSTTSTSSSSSSSSSHTHHSSSSTSSSSSSHSSSTHTSSSHSSSSSSSSSSTD
6vsb_B                    SSSSHTD         TSSSSSSSSSSTTSSSSSSSSSTD      TSTSSSSSSHSSSTHTSSSHSTSSSTD
6vsb_C                    SSSSHTD         TSSSSSSSSSSTTSTSSSSSSSSSHSHHSSSSTSSSSSSHSSSTHTSSSHSSSSSSSSSTD
6vxx_A                    STD            THSSSSSSSSSSTTSSSSSSSSSSSTSHHSSSSTSSSSSSHSSSTHTSSSHSSSSSSSSSTD
6vxx_B                    STD            THSSSSSSSSSSTTSSSSSSSSSSSTSHHSSSSTSSSSSSHSSSTHTSSSHSSSSSSSSSTD
6vxx_C                    STD            THSSSSSSSSSSTTSSSSSSSSSSSTSHHSSSSTSSSSSSHSSSTHTSSSHSSSSSSSSSTD
6vyb_A                    STD            THSSSSSSSSSSTTSSSSSSSSSSSTSHHSSSSTSSSSSSHSSSTHTSSSHSSSSSTD
6vyb_B                    STD            THSSSSSSSSTD  TSSSSSSSSSSTD  TSSSTSSSSSSHSSSTHTSSSHSSSSSTD
6vyb_C                    STD            THSSSSSSSSSSTTSSSSSSSSSSSTSHHSSSSTSSSSSSHSSSTHTSSSHSSSSSTD
6xs6_A                    STD            THSSSSSSTSSSTTSSSSSSSSSSSTSHHSSSTSSSSSSSHSSSTHTSSSHSSSSSSSSSTD
6xs6_B                    STD            THSSSSSSTSSSTTSSSSSSSSSSSTSHHSSSTSSSSSSSHSSSTHTSSSHSSSSSSSSSTD
6xs6_C                    STD            THSSSSSSTSSSTTSSSSSSSSSSSTSHHSSSTSSSSSSSHSSSTHTSSSHSSSSSSSSSTD
6xr8_A                    SSSSSSSSTSTHSHSSHSSSSSSSSTSTTSSSSSSSSSSSTSHHSSSSTSSSSSSHSSSTHTSSSHSSSSSSSSTD        TH
6xr8_B                    SSSSSSSSHSSSSTSSHSSSSSSSSSSSTTSSSSSSSSSSSHSHHSSSSTSSSSSSHSSSTHTSSSHSSSSSSSSTD        TH
6xr8_C                    SSSSSSSSHSTSSHSSHSSSSSSSSSSSTTSSSSSSSSSSSTSHTSSSSTSSSSSSHSSSTHTSSSHSSSSSSSSTD        TH
6xra_A
6xra_B
6xra_C
```

```
                          NTD                                                         RBD
                              260       270       280       290       300       310       320       330       340
B.1.617.2                 SGWTAGAAAYYVGYLQPRTFLLKYNENGTITDAVDCALDPLSETKCTLKSFTVEKGIYQTSNFRVQPTESIVRFPNITNLCPFGE
SSSCPred200               HHSHHTHSSSSSSSSSSHSSSSSSHHTSHSSHSTTSSSHHSHHHSSSHSSSSSSTTSSSSSSSSSSHSSSSSSSSHSSTSSHHHH
SSSCPred100               SHSSHTSSSSSSTHSSSSSSSSSSSHTSSHSSSTSSSSSHSHSHHHSHSSSSSSSTSSSSHHSSSSHSSSSSSSSSHSSSSTHHTS
SSSCPred                  HTSHHHSSSSSSHHSSSSSSSSSSHHTSSHHSSSHSSSHHHHSSSSSHSSSSSSSTSSSSSHSSSSSHHSSSSSSSSSSSSSHHH
B.1.617.1                 SGWTAGAAAYYVGYLQPRTFLLKYNENGTITDAVDCALDPLSETKCTLKSFTVEKGIYQTSNFRVQPTESIVRFPNITNLCPFGE
SSSCPred200               HHSHHTHSSSSSSSSSSHSSSSSSHHTSHSSHSTTSSSHHSHHHSSSHSSSSSSTTSSSSSSSSSSHSSSSSSSSHSSTSSHHHH
SSSCPred100               SHSSHTSSSSSSTHSSSSSSSSSSSHTSSHSSSTSSSSSHSHSHHHSHSSSSSSSTSSSSHHSSSSHSSSSSSSSSHSSSSTHHTS
SSSCPred                  HTSHHHSSSSSSHHSSSSSSSSSSHHTSSHHSSSHSSSHHHHSSSSSHSSSSSSSTSSSSSHSSSSSHHSSSSSSSSSSSSSHHH
B.1.427/429               SGWTAGAAAYYVGYLQPRTFLLKYNENGTITDAVDCALDPLSETKCTLKSFTVEKGIYQTSNFRVQPTESIVRFPNITNLCPFGE
SSSCPred200               HHSHHTHSSSSSSSSSSHSSSSSSHHTSHSSHSTTSSSHHSHHHSSSHSSSSSSTTSSSSSSSSSSHSSSSSSSSHSSTSSHHHH
SSSCPred100               SHSSHTSSSSSSTHSSSSSSSSSSSHTSSHSSSTSSSSSHSHSHHHSHSSSSSSSTSSSSHHSSSSHSSSSSSSSSHSSSSTHHTS
SSSCPred                  HTSHHHSSSSSSHHSSSSSSSSSSHHTSSHHSSSHSSSHHHHSSSSSHSSSSSSSTSSSSSHSSSSSHHSSSSSSSSSSSSSHHH
P.1                       SGWTAGAAAYYVGYLQPRTFLLKYNENGTITDAVDCALDPLSETKCTLKSFTVEKGIYQTSNFRVQPTESIVRFPNITNLCPFGE
SSSCPred200               HHSHHTHSSSSSSSSSSHSSSSSSHHTSHSSHSTTSSSHHSHHHSSSHSSSSSSTTSSSSSSSSSSHSSSSSSSSHSSTSSHHHH
SSSCPred100               SHSSHTSSSSSSTHSSSSSSSSSSSHTSSHSSSTSSSSSHSHSHHHSHSSSSSSSTSSSSHHSSSSHSSSSSSSSSHSSSSTHHTS
SSSCPred                  HTSHHHSSSSSSHHSSSSSSSSSSHHTSSHHSSSHSSSHHHHSSSSSHSSSSSSSTSSSSSHSSSSSHHSSSSSSSSSSSSSHHH
B.1.351                   SGWTAGAAAYYVGYLQPRTFLLKYNENGTITDAVDCALDPLSETKCTLKSFTVEKGIYQTSNFRVQPTESIVRFPNITNLCPFGE
SSSCPred200               HHSHHTHSSSSSSSSSSHSSSSSSHHTSHSSHSTTSSSHHSHHHSSSHSSSSSSTTSSSSSSSSSSHSSSSSSSSHSSTSSHHHH
SSSCPred100               HHHSHTSSSSSSTHSSSSSSSSSSSHTSSHSSSTSSSSSHSHSHHHSHSSSSSSSTSSSSHHSSSSHSSSSSSSSSHSSSSTHHTS
SSSCPred                  HTSHHHHSSSSSHHSSSSSSSSSSHHTSSHHSSSHSSSHHHHSSSSSHSSSSSSSTSSSSSHSSSSSHHSSSSSSSSSSSSSHHH
B.1.1.7                   SGWTAGAAAYYVGYLQPRTFLLKYNENGTITDAVDCALDPLSETKCTLKSFTVEKGIYQTSNFRVQPTESIVRFPNITNLCPFGE
SSSCPred200               HHSSHTHSSSSSSSSSSHSSSSSSHHTSSSSHSTTSSSHHSHHHSSSHSSSSSSTTSSSSSSSSSSHSSSSSSSSHSSTSSHHHH
SSSCPred100               HHSSHTSSSSSSTHSSSSSSSSSSSHTSSHSSSTSSSSSHSHSHHHSHSSSSSSSTSSSSHHSSSSHSSSSSSSSSHSSSSTHHTS
SSSCPred                  HTSHHHHSSSSSHHSSSSSSSSSSHHTSSHHSSSHSSSHHHHSSSSSHSSSSSSSTSSSSSHSSSSSHHSSSSSSSSSSSSSHHH
SARS-CoV-2          256   SGWTAGAAAYYVGYLQPRTFLLKYNENGTITDAVDCALDPLSETKCTLKSFTVEKGIYQTSNFRVQPTESIVRFPNITNLCPFGE     340
SSSCPred200               HHSHHTHSSSSSSSSSSHSSSSSSHHTSHSSHSTTSSSHHSHHHSSSHSSSSSSTTSSSSSSSSSSHSSSSSSSSHSSTSSHHHH
SSSCPred100               SHSSHTSSSSSSTHSSSSSSSSSSSHTSSHSSSTSSSSSHSHSHHHSHSSSSSSSTSSSSHHSSSSHSSSSSSSSSHSSSSTHHTS
SSSCPred                  HTSHHHSSSSSSHHSSSSSSSSSSHHTSSHHSSSHSSSHHHHSSSSSHSSSSSSSTSSSSSHSSSSSHHSSSSSSSSSSSSSHHH
6vsb_A                         TSSSSSSSSSSTSSSSSSSHHTSSHSSSSHHHSHHHHHHHHHTSHSSSSTSSSHSSSSSSSSSTSSSTD     TSTHHH
6vsb_B                           TSSSSSSSSTSSSSSSSHHTSSHSSSSHHHSHHHHHHHHHTSHSSSSTSSSHSSSSSSSSSTSSSSSSHSSSSSTHHH
6vsb_C                         TSSSSSSSSSSTSSSSSSSHHTSSHSSSSHHHSHHHHHHHHHTSHSSSSTSSSHSSSSSSSSSTSSSSSSHSTSSSTHHH
6m17_E                                                                                          THHHH
6m17_F                                                                                          THHHH
6vxx_A                           TSSSSSSSSTSSSSSSSHHTSSHSSSSHHHSHHHHHHHHHTSHSSSSTSSSHSSSSSSSSHSSSSSSSTSHSSSTHHH
6vxx_B                           TSSSSSSSSTSSSSSSSHHTSSHSSSSHHHSHHHHHHHHHTSHSSSSTSSSHSSSSSSSSHSSSSSSSTSHSSSTHHH
6vxx_C                           TSSSSSSSSTSSSSSSSHHTSSHSSSSHHHSHHHHHHHHHTSHSSSSTSSSHSSSSSSSSHSSSSSSSTSHSSSTHHH
6vyb_A                           TSSSSSSSSTSSSSSSSHHTSSHSSSSHHHSHHHHHHHHHTSHSSSSTSSSHSSSSSSSSHSSSSSSSTSHSSSTHHH
6vyb_B                           TSSSSSSSSTSSSSSSSHHTSSHSSSSHHHSHHHHHHHHHTSHSSSSTSSSHSSSSSSSSHSSSSSSSSHSTSSTHHH
6vyb_C                            TSSSSSSSTSSSSSSSHHTSSHSSSSHHHSHHHHHHHHHTSHSSSSTSSSHSSSSSSSSHSSSSSSSTSHSSSTHHH
6xr8_A                    STSSSSTSSSSSSSSSSSSSSSSSHHTSSHSSSSHHHSHHHHHHHHHTSHSSSSTSSSSSSSSSSSSHSSSSSSSSSHSSSTHHH
6xr8_B                    STSSSSSSSSSSSSSSSSSSSSSSHHTSSHSSSSHHHSHHHHHHHHHTSHSSSSTSSSSSSSSSSSSHSSSSSSSSSHSSSTHHH
6xr8_C                    STSSSSSSSSSSSSSSSSSSSSSSHHTSSHSSSSHHHSHHHHHHHHHTSHSSSSTSSSSSSSSSSSSHSSSSSSSSSHSSSTHHH
6xra_A
6xra_B
6xra_C
```

		RBD	
		341 350 360 370 380 390 400 410 420	
B.1.617.2		VFNATRFASVYAWNRKRISNCVADYSVLYNSASFSTFKCYGVSPTKLNDLCFTNVYADSFVIRGDEVRQIAPGQTGKIADYNYKL	
SSSCPred200		HHHHHHHHHHHHHHHHHHHHTSSSHHHHHHHHHHHHHHSSSSTSSSHSHHHHSSSTSSSSSSSSSTHSSSSSSSTSSTSSTSSSSSS	
SSSCPred100		SSSSSHHHSHHHHSHHHHHHSSSSHHHHHHSHHSHHSSSSSSSSSHSTHHSSSSSSSSSSSSSSSHHHHHSSSSTSTSSHHHHHSSS	
SSSCPred		HHHHHSSSSSSTHHHTSSSTSSHHTTHHSHSSHSTSSSSSTSSSSSSSSSHHSSHHSSSSSSSSHHTSSSSSSTSSTSSSSSHSSS	
B.1.617.1		VFNATRFASVYAWNRKRISNCVADYSVLYNSASFSTFKCYGVSPTKLNDLCFTNVYADSFVIRGDEVRQIAPGQTGKIADYNYKL	
SSSCPred200		HHHHHHHHHHHHHHHHHHHHTSSSHHHHHHHHHHHHHSSSSSTSSHHSHHTHSSSTSSSSSSSSSTHSSSSSSSTSSTSSTSSSSSS	
SSSCPred100		SSSSSHHHSHHHHSHHHHHHSSSSHHHHHHSHHSHHSSSSSSSSSHSTHHSSSSSSSSSSSSSSSHHHHHSSSSTSTSSSHHHHSSS	
SSSCPred		HHHHHSSSSSSTHHHTSSSTSSTHTTHHSTSSHSTSSSSSTSSSSSSSSSHHSSHHSSSSSSSSHHTSSSSSSTSSTSSSSSHSSS	
B.1.427/429		VFNATRFASVYAWNRKRISNCVADYSVLYNSASFSTFKCYGVSPTKLNDLCFTNVYADSFVIRGDEVRQIAPGQTGKIADYNYKL	
SSSCPred200		HHHHHHHHHHHHHHHHHHHHTSSSHHHHHHHHHHHHHSSSSSTSSHHSSHTHSSSTSSSSSSSSSTHSSSSSSSTSSTSSTSSSSSS	
SSSCPred100		SSSSSHHHSHHHHSHHHHHHSSSSHHHHHHSHHSHHSSSSSSSSSHSTHHSSSSSSSSSSSSSSSHHHHHSSSSTSTSSSHHHHSSS	
SSSCPred		HHHHHSSSSSSTHHHTSSSTSSTHTTHHSTSSHSTSSSSSTSSSSSSSSSHHSSHHSSSSSSSSHHTSSSSSSTSSTSSSSSHSSS	
P.1		VFNATRFASVYAWNRKRISNCVADYSVLYNSASFSTFKCYGVSPTKLNDLCFTNVYADSFVIRGDEVRQIAPGQTGTIADYNYKL	
SSSCPred200		HHHHHHHHHHHHHHHHHHHHTSSSHHHHHHHHHHHHHSSSSSTSSHSSSHTHSSSTSSSSSSSSSTHSSSSSSSTSSTSHHHHHSTS	
SSSCPred100		SSSSSHHHSHHHHSHHHHHHSSSSHHHHHHSHHSHHSSSSSSSSSHSTHHSSSSSSSSSSSSSSSHHHHSHSSTSSTSHHHHHSSS	
SSSCPred		HHHHHSSSSSSTHHHTSSSTSSTHTTHHSTSSHSTSSSSSTSSSSSSSSSHHSSHHSSSSSSSSHHHHHSSSTSSTSHHHHHTTS	
B.1.351		VFNATRFASVYAWNRKRISNCVADYSVLYNSASFSTFKCYGVSPTKLNDLCFTNVYADSFVIRGDEVRQIAPGQTGNIADYNYKL	
SSSCPred200		HHHHHHHHHHHHHHHHHHHHTSSSHHHHHHHHHHHHHSSSSSTSSHSSSHTHSSSTSSSSSSSSSTHSSSSSSHTSTHSSSSSSSSS	
SSSCPred100		SSSSSHHHSHHHHSHHHHHHSSSSHHHHHHSHHSHHSSSSSSSSSHSTHHSSSSSSSSSSSSSSSHHHSSHSSTSSSSHHHHHSSS	
SSSCPred		HHHHHSSSSSSTHHHTSSSTSSTHTTHHSTSSHSTSSSSSTSSSSSSSSSHHSSHHSSSSSSSSHHHHHSSSTSSTTHHHHHSSS	
B.1.1.7		VFNATRFASVYAWNRKRISNCVADYSVLYNSASFSTFKCYGVSPTKLNDLCFTNVYADSFVIRGDEVRQIAPGQTGKIADYNYKL	
SSSCPred200		HHHHHHHHHHHHHHHHHHHHTSSSHHHHHHHHHHHHHHSSSSTSSHHSHHHHSSSTSSSSSSSSSTHSSSSSSSTSSTSSTSSSSSS	
SSSCPred100		SSSSSHHHSHHHHSHHHHHHSSSSHHHHHHSHHSHHSSSSSSSSSHSTHHSSSSSSSSSSSSSSSHHHHHSSSSTSTSSSHHHHSSS	
SSSCPred		HHHHHSSSSSSTHHHTSSSTSSTHTTHHHSSSSSTSSSSSTSSSSSSSSSHHSSHHSSSSSSSSTHTSSSSSSTSSTSSSSSHSSS	
SARS-CoV-2	341	VFNATRFASVYAWNRKRISNCVADYSVLYNSASFSTFKCYGVSPTKLNDLCFTNVYADSFVIRGDEVRQIAPGQTGKIADYNYKL	425
SSSCPred200		HHHHHHHHHHHHHHHHHHHHTSSSHHHHHHHHHHHHHSSSSSTSSHSSSHTHSSSTSSSSSSSSSTHSSSSSSSTSSTSSTSSSSSS	
SSSCPred100		SSSSSHHHSHHHHSHHHHHHSSSSHHHHHHSHHSHHSSSSSSSSSHSTHHSSSSSSSSSSSSSSSHHHHHSSSSTSTSSSHHHHSSS	
SSSCPred		HHHHHSSSSSSTHHHTSSSTSSTHTTHHSTSSHSTSSSSSTSSSSSSSSSHHSSHHSSSSSSSSHHTSSSSSSTSSTSSSSSHSSS	
6vsb_A		HHHSHSSSSHHTSSSSSSSTSSSSHHHHHHSHHSHSSSSSTSSHHHHHHSSSHSSSSSSSSSSHHHHHHTSSTSSTHHHHHHSSS	
6vsb_B		HHHSHSSSSHHTSSSSSSSTSSSSHHHHHHSHHSHSTSSSTSSHHHHHHSSSHSSSSSSSSSSHHHHHHTSSTSSTHHHHHHSSS	
6vsb_C		HHTSHTSSSHHTSSSSSSSTSSSSHHHHHHSHHSHSSSSSTSSHHHHHHSSSHSSSSSSSSSSHHHHHHTSSTSSTHHHHHHSSS	
6m17_E		HHSSHSSSSHHSSSSSSSSSTSHSHTHHSTTSHHSHSSSSHHSSSTHTTSSHTSTSSSSSSSSSHTHHHHHSSTTSSHTTHHHSSS	
6m17_F		HHSSHSSSSHHSSSSSSSSSTSHSHTHHSTTSHHSHSSSSHHSSSTHTTSSHTSTSSSSSSSSSHTHHHHHSSTTSSHTTHHHSSS	
6vxx_A		HHHSHSSSSHHTSSSSSSSTSSSSHHHHHHSHHSHSTSSHHSSHHHHHHSSSHSSSSSSSSSSHHHHHHTSSTSSTHHHHHHSSS	
6vxx_B		HHHSHSSSSHHTSSSSSSSTSSSSHHHHHHSHHSHSTSSHHSSHHHHHHSSSHSSSSSSSSSSHHHHHHTSSTSSTHHHHHHSSS	
6vxx_C		HHHSHSSSSHHTSSSSSSSTSSSSHHHHHHSHHSHSTSSHHSSHHHHHHSSSHSSSSSSSSSSHHHHHHTSSTSSTHHHHHHSSS	
6vyb_A		HHHSHSSSSHHTSSSSSSSTSSSSHHHHHHSHHSHSSSSHHSSHHHHHHSSSHSSSSSSSSSSHHHHHHTSSTSSTHHHHHHSSS	
6vyb_B		HHHSHSSSSHHTSSSSSSSTSSSSHHHHHHSHHSHSSSSHHSSHHHHHHSSSHSSSSSSSSSSHHHHHHTSSTSSTHHHHHHSSS	
6vyb_C		HHHSHSSSSHHTSSSSSSSTSSSSHHHHHHSHHSHSSSSHHSSHHHHHHSSSHSSSSSSSSSSHHHHHHTSSTSSTHHHHHHSSS	
6xr8_A		HHHSHSSSSHHTSSSSSSSTSSTTHHHHHHHHHTSHSSSSHHSSHHHHHHSSSHSSSSSSSSSSHHHHHHHSSTSSTHHHHHHSSS	
6xr8_B		HHHSHSSSSHHTSSSSSSSTSSTTHHHHHHHHHTSHSSSSHHSSHHHHHHSSSHSSSSSSSSSSHHHHHHHSSTSSTHHHHHHSSS	
6xr8_C		HHHSHSSSSHHTSSSSSSSTSSTTHHHHHHHHHTSHSSSSHHSSHHHHHHSSSHSSSSSSSSSSHHHHHHHSSTSSTHHHHHHSSS	
6xra_A			
6xra_B			
6xra_C			

```
                      RBD        RBM
                          430       440       450       460       470       480       490       500       510
B.1.617.2             PDDFTGCVIAWNSNNLDSKVGGNYNYRYRLFRKSNLKPFERDISTEIYQAGSKPCNGVEGFNCYFPLQSYGFQPTNGVGYQPYRV
SSSCPred200           SHHSHTSSSSSSHHHHHHHHTSSHHHSSSSSSSSTSSSSSSSHHHHHHHSTSSHSSTSSTSSSSSSSSSSSSSSSHTSHSSSSSS
SSSCPred100           SHHTSSSSSSSSTSSHHSHSSHSHTSSSSSSSSHHSSHHHHHHHSHHSSSTSSSSSSSSTSSSSSSSSTHSSSHHSSSSHSSSSS
SSSCPred              SHHHSTSSSSSSSHSHSHHHSTSSSSSSSSSSHSHSSHHSHSHHSSSHSSTSSHHHTSSTSSSSSSSSTSTSSHHSTSTSSSSSS
B.1.617.1             PDDFTGCVIAWNSNNLDSKVGGNYNYRYRLFRKSNLKPFERDISTEIYQAGSTPCNGVQGFNCYFPLQSYGFQPTNGVGYQPYRV
SSSCPred200           SHHSHTSSSSSSHHHHHHHHTSSHHSSSSSSSSSTSSSSSSSSHSHHSSSTSHHSSTSTTSHSSSSSSSSSSSSSHTHHSSSSSS
SSSCPred100           SHHTSSSSSSSSTSSHHSHSSHSHTSSSSSSSSHHSSHHHHHHHHHHHHSTSTSHHTSSSSTSSSSSSTHSSSHHSSSSHSSSSS
SSSCPred              SHHHHTSSSSSSSHSHSSHHSTSSSSSSSSSSHHHSSHHSHSHHSSSSSSHSSHTHTSHTSSSSSTHSTSTSSHHSTSTSSSSSS
B.1.427/429           PDDFTGCVIAWNSNNLDSKVGGNYNYRYRLFRKSNLKPFERDISTEIYQAGSTPCNGVEGFNCYFPLQSYGFQPTNGVGYQPYRV
SSSCPred200           SHHSHTSSSSSSHHHHHHHHTSSHHSSSSSSSSSTSSSSSSSSHHHHSSSTSSHSHTHTTSSSSSSSSSSSSSSSHTHHSSSSSS
SSSCPred100           SHHTSSSSSSSSTSSHHSHSSHSHTSSSSSSSSHHSSHHHHHHHHHHHSSTSSSSTTSSTSSSSSSSSTHSSSHHSSSSHSSSSS
SSSCPred              SHHHHTSSSSSSSHSHSSHHSTSSSSSSSSSSHHHSSHHSHSHHSSSSSHHSSSSSTSSTSSSSSTSSTSTSSHHSTSTSSSSSS
P.1                   PDDFTGCVIAWNSNNLDSKVGGNYNYLYRLFRKSNLKPFERDISTEIYQAGSTPCNGVKGFNCYFPLQSYGFQPTYGVGYQPYRV
SSSCPred200           SHHSSTSSSSSSHHHHHHHHTTHHHSSSSSSSSSTSSSSSSSSHHSHHSSTSHHTHTSSSSSSSTSSSSSSSSSHSTHTSSTSSS
SSSCPred100           SHHTSSSSSSSSTSSHHHHSSHHHHHHHHHHHHHHSSHHHHHHHHHHHSSTSTSSHTSSTSSSSSSSSTHSSTHHSSHHHSSSSS
SSSCPred              SHHHHTSSSSSSHHSHHSHHHHSHHHHHHHHHHSHSSHHHHSHHSSSSSSHHSHHTTSTTSSSSSSSSTSTSSHHTTSSSSSSSS
B.1.351               PDDFTGCVIAWNSNNLDSKVGGNYNYLYRLFRKSNLKPFERDISTEIYQAGSTPCNGVKGFNCYFPLQSYGFQPTYGVGYQPYRV
SSSCPred200           SHHSHTSSSSSSHHHHSHHHTTHHHSSSSSSSSSTSSSSSSSSHHSHHSSTSHHTHTSSSSSSSTSSSSSSSSSHSTHTSSTSSS
SSSCPred100           SHHTSSSSSSSSTSSHHHHSSHHHHHHHHHHHHHHSSHHHHHHHHHHHSSTSTSSHTSSTSSSSSSSSTHSSTHHSSHHHSSSSS
SSSCPred              SHHHHTSSSSSSHHSHHSHHHHSHHHHHHHHHHSHSSHHHHSHHSSSSSSHHSHHTTSTTSSSSSSSSTSTSSHHTTSSSSSSSS
B.1.1.7               PDDFTGCVIAWNSNNLDSKVGGNYNYLYRLFRKSNLKPFERDISTEIYQAGSTPCNGVEGFNCYFPLQSYGFQPTYGVGYQPYRV
SSSCPred200           SHHSSTSSSSSSHHHHHHHHTTHHHSSSSSSSSSTSSSSSSSSHHHHSSSTSSHSHTHTTSSSSSSSSSSSSSSHSTHTSTTSSS
SSSCPred100           SHHTSSSSSSSSTSSHHHHSSHHHHHHHHHHHHHHSSHHHHHHHHHHHSSTSSSSTTSSTSSSSSSSSTHSSTHHSSHHHSSSSS
SSSCPred              SHHHHTSSSSSSHHSHHSHHHHSHHHHHHHHHHSHSSHHHHSHHSSSSSHHSSSSSTSSTSSSSSSSSTSTSSSSTTSSHSSSSS
S477N                 PDDFTGCVIAWNSNNLDSKVGGNYNYLYRLFRKSNLKPFERDISTEIYQAGNTPCNGVEGFNCYFPLQSYGFQPTNGVGYQPYRV
SSSCPred200           SHHSHTSSSSSSHHHHHHHHTTHHHSSSSSSSSSTSSSSSSTHHHHHSTTTSSHSHTSSTSSSSSSSSSSSSSSSHTHHSSSSSS
SSSCPred100           SHHTSSSSSSSSTSSHHHHSSHHHHHHHHHHHHHHSSHHHHHHHHHHHHSHSSHHSTSSTSSSSSSSSTHSSSHHSSSSHSSSSS
SSSCPred              SHHHHTSSSSSSHHSHHSHHHHSHHHHHHHHHHSHSSHHHHSHHSSSSSSTHSSSSTSSTSSSSSSSSTSTSSHHSTSTSSSSSS
SARS-CoV-2      426   PDDFTGCVIAWNSNNLDSKVGGNYNYLYRLFRKSNLKPFERDISTEIYQAGSTPCNGVEGFNCYFPLQSYGFQPTNGVGYQPYRV   510
SSSCPred200           SHHSHTSSSSSSHHHHHHHHTTHHHSSSSSSSSSTSSSSSSSSHHHHSSSTSSHSHTHTTSSSSSSSSSSSSSSSHTHHSSSSSS
SSSCPred100           SHHTSSSSSSSSTSSHHHHSSHHHHHHHHHHHHHHSSHHHHHHHHHHHSSTSSSSTTSSTSSSSSSSSTHSSSHHSSSSHSSSSS
SSSCPred              SHHHHTSSSSSSHHSHHSHHHHSHHHHHHHHHHSHSSHHHHSHHSSSSSHHSSSSSTSSTSSSSSTSSTSTSSHHSTSTSSSSSS
6vsb_A                STSSSHSSSSSSHHHHHTD    THSSSTD                                   TSSSSTSSHTD THHSSSSS
6vsb_B                STSSSHSSSSSSHHHHHTD    THSSSTD    TSSSTSSTHSSSSTD            TSSSHSSSSSSSHTD THHSSSSS
6vsb_C                STSSSHSSSSSSHHHHHTD    THSSSTD    TSSSTSSSHSSTD              TSSSHSSSSSSTD   THHSSSSS
6m17_E                STSHSHSSSSSSTHHHSTSHHTSTTSSSSHSSSTSSSSTSSSHSSSSHTSTSTSTSTTTHSTSSSHSSSHTSSHHSSHHHSSSSS
6m17_F                STSHSHSSSSSSTHHHSTSHHTSTTSSSSHSSSTSSSSTSSSHSSSSHTSTSTSTSTTTHSTSSSHSSSHTSSHHSSHHHSSSSS
6vxx_A                SHHSSTSSSSSSTHHHHSTD TSHHSSSTD      TSTSSTTD                   TSHSSSSSSSHHTDTHHSSSSS
6vxx_B                SHHSSTSSSSSSTHHHHSTD TSHHSSSTD      TSTSSTTD                   TSHSSSSSSSHHTDTHHSSSSS
6vxx_C                SHHSSTSSSSSSTHHHHSTD TSHHSSSTD      TSTSSTTD                   TSHSSSSSSSHHTDTHHSSSSS
6vyb_A                SHHSSTSSSSSSHHHHTD    THHSSSSHSSHSSSSSTSSSTSTD                  THSSSSTSSHHTDTHHSSSSS
6vyb_B                SHHSSTSSSSSSTHHHHSSHHTSHHSSSTD      TSTSTD                       TSSSSTSSHHSSHHHSSSSS
6vyb_C                SHHSSTSSSSSSTHHHHSTDTHHSSSTD        TSTSSSHSTD                   TSSSSTSSHHSSHHHSSSSS
6xs6_A
6xs6_B
6xs6_C
6xr8_A                SHTSSTSSSSSSTHHHHSSHHTSHHSSSSHSSHSSSSSTSSSHSHSSSSHSHSSSSTHSTSTSSSHSSSSTTSHHSSSTHSSSSS
6xr8_B                SHHSSTSSSSSSTHHHHSSHHTSHHSSSSHSSHSSSSSTSSSHSHSSSSHSHSSSSTHSTSTSSSHSSSSTTSHHSSSTHSSSSS
6xr8_C                SHTSSTSSSSSSTHHHHSSHHTSHHSSSSHSSHSSSSSTSSSHSHSSSSHSHSSSSTHSSSTSSSHSSSSTSSHHSSSTHSSSSS
6xra_A
6xra_B
6xra_C
```

		RBD SD1 SD2	
		511 520 530 540 550 560 570 580 590	
B.1.617.2		VVLSFELLHAPATVCGPKKSTNLVKNKCVNFNFNGLTGTGVLTESNKKFLPFQQFGRDIADTTDAVRDPQTLEILDITPCSFGGV	
SSSCPred200		SSSSSSSSTSSSSSSSHHHSSSHHHHSSSSSSSSSTSSSSSSSSSTTSSSSHHSHHSSHHHHHHHHHSSSHHHTSSSSSSSTSSTSS	
SSSCPred100		SSSSSSSSSTSHHHSSSHHHSSSHSHSSSSSSSSSSSSSTSTHSSHHTSSSSSSSSSTHHHHSHHHHHHSHHSSSSSSSSSSSSSTTS	
SSSCPred		SSSSSSSHSSSSSHHHHHHSSSSSSSSSSSSSSSSTSSTSSSSSHHHTSHSSSSSSSSHSHHTSHSSSHSHHHHSSHSSSSSSSSTTS	
B.1.617.1		VVLSFELLHAPATVCGPKKSTNLVKNKCVNFNFNGLTGTGVLTESNKKFLPFQQFGRDIADTTDAVRDPQTLEILDITPCSFGGV	
SSSCPred200		SSSSSSSSTSSSSSSSSHHSSSHHHHSSSSSSSSSTSSTSSSSSSSTTSSSSHHSSSSSHHHHHHHHHSSSHHHTSSSSSSSSSSTSS	
SSSCPred100		SSSSSSSSSTSHHHSSSHHHSSSHSHSSSSSSSSSSSSSTSTHSSHHTSSSSSSSSTTHHHHSHHHHHHSHHSSSSSSSSSSSSSTTS	
SSSCPred		SSSSSSSHSSSSSHHHHHHSSSSSSSSSHSSSSSSSTSSTSSSSSHHHTSHHSSSSSSSHSHHHSHSSSHSHHHHSSHSSSSSSSSTTS	
B.1.427/429		VVLSFELLHAPATVCGPKKSTNLVKNKCVNFNFNGLTGTGVLTESNKKFLPFQQFGRDIADTTDAVRDPQTLEILDITPCSFGGV	
SSSCPred200		SSSSSSSSTSSSSSSSSHHSSSHHHHSSSSSSSSSTSSTSSSSSSSTTSSSSHHSSSSSHHHHHHHHHSSSHHHTSSHSSSSSSSSTSS	
SSSCPred100		SSSSSSSSSTSHHHSSSHHHSSSHSHSSSSSSSSSSSSSTSTHSSHHTSSSSSSSSTTHHHHSHHHHHHSHHSSSSSSSSSSSSSTTS	
SSSCPred		SSSSSSSHSSSSSHHHHHHSSSSSSSSSHSSSSSSSTSSTSSSSSHHHTSHHSSSSSSSHSHHHSHSSSHSHHHHSSHSSSSSSSSTTS	
P.1		VVLSFELLHAPATVCGPKKSTNLVKNKCVNFNFNGLTGTGVLTESNKKFLPFQQFGRDIADTTDAVRDPQTLEILDITPCSFGGV	
SSSCPred200		SSSSSSSSTSSSSSSSSHHSSSHHHHSSSSSSSSSTSSTSSSSSSSTTSSSSHHSSSSSHSHHHHHHHSSSHHHTSSHSSSSSSSSTSS	
SSSCPred100		SSSSSSSSSTSHHHSSSHHHSSSHSHSSSSSSSSSSSSSTSTHSSHHTSSSSSSSSTTHHHHSHHHHHHSHHSSSSSSSSSSSSSTTS	
SSSCPred		SSSSSSSHSSSSSHHHHHHSSSSSSSSSHSSSSSSSTSSTSSSSSHHHTSHHSSSSSSSHSHHHSHSSSHSHHHHSSHSSSSSSSSTTS	
B.1.351		VVLSFELLHAPATVCGPKKSTNLVKNKCVNFNFNGLTGTGVLTESNKKFLPFQQFGRDIADTTDAVRDPQTLEILDITPCSFGGV	
SSSCPred200		SSSSSSSSTSSSSSSSSHHSSSHHHHSSSSSSSSSTSSTSSSSSSSTTSSSSHHSSSSSHSHHHHHHHSSSHHHTSSHSSSSSSSSTSS	
SSSCPred100		SSSSSSSSSTSHHHSSSHHHSSSHSHSSSSSSSSSSSSSTSTHSSHHTSSSSSSSSTTHHHHSHHHHHHSHHSSSSSSSSSSSSSTTS	
SSSCPred		SSSSSSSHSSSSSHHHHHHSSSSSSSSSHSSSSSSSTSSTSSSSSHHHTSHHSSSSSSSHSHHHSHSSSHSHHHHSSHSSSSSSSSTTS	
B.1.1.7		VVLSFELLHAPATVCGPKKSTNLVKNKCVNFNFNGLTGTGVLTESNKKFLPFQQFGRDIDDTTDAVRDPQTLEILDITPCSFGGV	
SSSCPred200		SSSSSSSSTSSSSSSSHHHSSSHHHHHSSSSSSSSTSSTSSSSSSSTTSSSSHSSSSTSSSSHHHHSSSSHHHTSSHSSSSSSSSTSS	
SSSCPred100		SSSSSSSSSTSHHHSSSHHHSSSHSHSSSSSSSSSSSSSTSTHSSHHTSSSSSSSSTTHHHHSHHHHHHSHHSSSSSSSSSSSSSTTS	
SSSCPred		SSSSSSSHSSSSSHHHHHHSSSSSSSSSSSSSSSSTSSTSSSSSHHHTSHHHHHSSSSSHHTSSSSSSSHHHHSSHSSSSSSSSTTS	
SARS-CoV-2	511	VVLSFELLHAPATVCGPKKSTNLVKNKCVNFNFNGLTGTGVLTESNKKFLPFQQFGRDIADTTDAVRDPQTLEILDITPCSFGGV	595
SSSCPred200		SSSSSSSSTSSSSSSSSHHSSSHHHHSSSSSSSSSTSSTSSSSSSSTTSSSSHHSSSSSHHHHHHHHHSSSHHHTSSHSSSSSSSSTSS	
SSSCPred100		SSSSSSSSSTSHHHSSSHHHSSSHSHSSSSSSSSSSSSSTSTHSSHHTSSSSSSSSTTHHHHSHHHHHHSHHSSSSSSSSSSSSSTTS	
SSSCPred		SSSSSSSHSSSSSHHHHHHSSSSSSSSSHSSSSSSSTSSTSSSSSHHHTSHHSSSSSSSHSHHHSHSSSHSHHHHSSHSSSSSSSSTTS	
6vsb_A		SSSSSSSTHSSSHSSSSSTSSSSSSSTSSSSSSTTTSSSSTSSSSSHSSSSHTSHSSSHSTTSSHSSSSHHHTSSSSSSSSSSSTSS	
6vsb_B		SSSSSSSTHSSSHSSSTSHTSSSSSSTSSSSSTTTTSSTSTSSSSSHSSSSHTSHSSSHSTTSSHSSSSHHHTSSSSTSSSSSSTSS	
6vsb_C		SSSSSSSTHSSSHSSSSSSSSSSSSSTSSSSSSSSTTSSSSTSSSSSHSSSSHTSHSSSHSTTSSHSSSSHHHTSSSSSSSSSSSTSS	
6m17_E		SSSSSSSTD	
6m17_F		SSSSSSSTD	
6vxx_A		SSSSSSHSSSSSHSSSTSSSSSSSSSTSSSSSSSTTSSTSTSSSSSHSTSSHHSHSSSSHHTSSHSSSSHHSTSSSSSSSSSSSTSS	
6vxx_B		SSSSSSHSSSSSHSSSTSSSSSSSSSTSSSSSSSTTSSTSTSSSSSHSTSSHHSHSSSSHHTSSHSSSSHHSTSSSSSSSSSSSTSS	
6vxx_C		SSSSSSHSSSSSHSSSTSSSSSSSSSTSSSSSSSTTSSTSTSSSSSHSTSSHHSHSSSSHHTSSHSSSSHHSTSSSSSSSSSSSTSS	
6vyb_A		SSSSSSHSSSSSHSSSTSSSSSSSSSTSSSSSSSTTSSTSTSSSSSHSTSSHHSHSSSSHHTSSHSSSSHHSTSSSSSSSSSSSTSS	
6vyb_B		SSSSTD THSSSSSSSSSSSSTSSSSSSSTHSSTSTSSSSSHSSSSHHSHSSSSHTTSSHSSSSHHSTSSSSSSSSSSSTSS	
6vyb_C		SSSSSSSSHSSSHSSSTSSSSSSSSSTSSSSSSSTTSSTSTSSSSSHSSSSHHSHSSSSHHTSSHSSSSHHSTSSSSSSSSSSSTSS	
6xs6_A		TSSSSTSSSSSSSTTSSTSTSTSSSTSSSSHHSHSSSSHTTSSHSSSSHHSSSSSSSSSSSSSTSS	
6xs6_B		TSSSSTSSSSSSSTTSSTSTSTSSSTSSSSHHSHSSSSHHTSSHSSSSHHSTSSSSSSSSSSSTSS	
6xs6_C		TSSSSTSSSSSSSTTSSTSTSTSSSTSSTSHHSHSSSSHHTSSHSSSSHHSTSSSSSSSSSSSTSS	
6xr8_A		SSSSSSSTHSSSHSSSTSSSSSSSSSTSSSSSSSTTTSSSTSSSSSHSSSSHHSHSSSSHHTSSHSSSSHHSSSSSSSSSSHSSTSS	
6xr8_B		SSSSSSSTHSSSHSSSTSSSSSSSSSTSSSSSSSTTTSSSTSSSSSHSTSSHHSHSSSSHHTSSHSSSSHHSTSSSSSSSHSSTSS	
6xr8_C		SSSSSSSTHSSSHSSSTSSSSSSSSSTSSSSSSSTTTSTSTSSSSSHSTSSHHSHSSSSHHTSSHSSSSHHSTSSSSSSSHSSTSS	
6xra_A			
6xra_B			
6xra_C			

```
                   SD2
                        600       610       620       630       640       650       660       670       680
B.1.617.2          SVITPGTNTSNQVAVLYQGVNCTEVPVAIHADQLTPTWRVYSTGSNVFQTRAGCLIGAEHVNNSYECDIPIGAGICASYQTQTNS
SSSCPred200        SSSSSTHHSTTSSSSSSSTSSSHHSHSSSTSSHHSSSSSSSSSSTSHSSSSHHTSSSSSSSSSHHSSSHSSHTTTHSHSSSSHHSS
SSSCPred100        SSSSHSSSHSHSSSSSSSTSSSSHSSSSSSHHHSSSSSSSSSSSTSHSSSSSSSSSSSTSHSSSSSSSSHSSSSTTSSHSSSHHSHS
SSSCPred           SSSSSHSSHSSSSSSSSSTSSHTSSSSSSSTHHHSSSSSSSSSHHHSSSSSSSSSSSSTSSSSTSSSSSHSSSTSTHSSSSSSSSSHS
B.1.617.1          SVITPGTNTSNQVAVLYQGVNCTEVPVAIHADQLTPTWRVYSTGSNVFQTRAGCLIGAEHVNNSYECDIPIGAGICASYQTQTNS
SSSCPred200        SSSSSTHHSTTSSSSSSSTSSSHHSHSSSHSSHHSSSSSSSSSSTSHSSSSHHTSSSSSSSSSHHSSSHSSHTTTHSHSSSSHHSS
SSSCPred100        SSSSHSSSHSHSSSSSSSTSSSSHSSSSSSHHHSSSSSSSSSSSTSHSSSSSSSSSSSTSHSSSSSSSSHSSSSTTSSHSSSHHSHS
SSSCPred           SSSSSHSSHSSSSSSSSSTSSHTSSSSSSSTHHHSSSSSSSSSHHHSSSSSSSSSSSSTSSSSTSSSSSHSSSTSTHSSSSSSSSSHS
B.1.427/429        SVITPGTNTSNQVAVLYQGVNCTEVPVAIHADQLTPTWRVYSTGSNVFQTRAGCLIGAEHVNNSYECDIPIGAGICASYQTQTNS
SSSCPred200        SSSSSTHHSTTSSSSSSSTSSSHHSHSSSHSSHHSSSSSSSSSSTSHSSSSHHTSSSSSSSSSHHSSSHSSHTTTHSHSSHSHHSS
SSSCPred100        SSSSHSSSHSHSSSSSSSTSSSSHSSSSSSHHHSSSSSSSSSSSTSHSSSSSSSSSSSTSHSSSSSSSSHSSSSTTSSSSSSHHSHS
SSSCPred           SSSSSHSSHSSSSSSSSSTSSHTSSSSSSSTHHHSSSSSSSSSHHHSSSSSSSSSSSSTSSSSTSSSSSHSSSTSTHSSSSSSSSSHS
P.1                SVITPGTNTSNQVAVLYQGVNCTEVPVAIHADQLTPTWRVYSTGSNVFQTRAGCLIGAEYVNNSYECDIPIGAGICASYQTQTNS
SSSCPred200        SSSSSTHHSTTSSSSSSSTSSSHHSHSSSHSSHHSSSSSSSSSSTSHSSSSHHTSSSSSSSSSSHSSSSHSSHTTTHSHSSHSHHSS
SSSCPred100        SSSSHSSSHSHSSSSSSSTSSSSHSSSSSSHHHSSSSSSSSSSSTSHSSSSSSSSSSSSSSHSSSTSHHSHSSSSTTSSSSSSHHSHS
SSSCPred           SSSSSHSSHSSSSSSSSSTSSHTSSSSSSSTHHHSSSSSSSSSHHHSSSSSSSSSSSSSSSSSSTTSSSSHSSSTSTHSSSSSSSSSHS
B.1.351            SVITPGTNTSNQVAVLYQGVNCTEVPVAIHADQLTPTWRVYSTGSNVFQTRAGCLIGAEHVNNSYECDIPIGAGICASYQTQTNS
SSSCPred200        SSSSSTHHSTTSSSSSSSTSSSHHSHSSSHSSHHSSSSSSSSSSTSHSSSSHHTSSSSSSSSSHHSSSHSSHTTTHSHSSHSHHSS
SSSCPred100        SSSSHSSSHSHSSSSSSSTSSSSHSSSSSSHHHSSSSSSSSSSSTSHSSSSSSSSSSSTSHSSSSSSSSHSSSSTTSSSSSSHHSHS
SSSCPred           SSSSSHSSHSSSSSSSSSTSSHTSSSSSSSTHHHSSSSSSSSSHHHSSSSSSSSSSSSTSSSSTSSSSSHSSSTSTHSSSSSSSSSHS
B.1.1.7            SVITPGTNTSNQVAVLYQGVNCTEVPVAIHADQLTPTWRVYSTGSNVFQTRAGCLIGAEHVNNSYECDIPIGAGICASYQTQTNS
SSSCPred200        SSSSSTHHHTTSSSSSSSTSSSHHSHSSSHSSHHSSSSSSSSSSTSHSSSSHHTSSSSSSSSSHHSSSHSSHTTTHSHSSSSTHHH
SSSCPred100        SSSSHSSSHSHSSSSSSSTSSSSHSSSSSSHHHSSSSSSSSSSSTSHSSSSSSSSSSSTSHSSSSSSSSHSSSSTTSSSSSSSHHHH
SSSCPred           SSSSSHSSHSSSSSSSSSTSSHTSSSSSSSTHHHSSSSSSSSSHHHSSSSSSSSSSSSTSSSSTSSSSSHSSSTSTHSSSSSSSSSSH
SARS-CoV-2    596  SVITPGTNTSNQVAVLYQDVNCTEVPVAIHADQLTPTWRVYSTGSNVFQTRAGCLIGAEHVNNSYECDIPIGAGICASYQTQTNS  680
SSSCPred200        SSSSSTHHSHHHSSSSSSTSSSHSSSSSSSTSSHHSSSSSSSSSSTSHSSSSHHTSSSSSSSSSHHSSSHSSHTTTHSHSSHSHHSS
SSSCPred100        SSSSHSSHHSHSSSSSSSSSTSSHSSSSSSHHHSSSSSSSSSSSTSHSSSSSSSSSSSTSHSSSSSSSSHSSSSTTSSSSSSHHSHS
SSSCPred           SSSSHHSSSSSHHHHSSSSHHHSSSSSSSSSTHHHSSSSSSSSSHHHSSSSSSSSSSSSTSSSSTSSSSSHSSSTSTHSSSSSSSSSHS
6vsb_A             SSSSHTHHHSHSSSSSSSTSSTHSTD                  TSSSSSHHTSSSTSSSSHSSSSSHSSHTTTSSTD
6vsb_B             SSSSHTHHHSHSSSSSSSTSSHHSTD                TTHSSSSSHHTSSSTSSSSHSSSSSHSSHTTTSSTD
6vsb_C             SSSSHTHHHSHSSSSSSSTSSTHSTD                   TSSSSHHTSSSTSSSSHSSSSSHSSHTTTSSTD
6vxx_A             SSSSSSHHHSHSSSSSSSTSSTHHTD                   TSSSSHHTSSSTSSSSHSSSSSHSSHTTTSSSSSSTD
6vxx_B             SSSSSSHHHSHSSSSSSSTSSTHHTD                   TSSSSHHTSSSTSSSSHSSSSSHSSHTTTSSSSSSTD
6vxx_C             SSSSSSHHHSHSSSSSSSTSSTHHTD                   TSSSSHHTSSSTSSSSHSSSSSHSSHTTTSSSSSSTD
6vyb_A             SSSSSSHHHSHSSSSSSSTSSTHHTD                   TSSSSHHTSSSTSSSSHSSSSSHSSHTTTSSSSSSTD
6vyb_B             SSSSSSHHHSHSSSSSSSTSSHHHTD                   TSSSSHHTSSSTSSSSHSSSSSHSSHTTTSSSSSSTD
6vyb_C             SSSSHTHHHSHSSSSSSSTSSHHHTD                   TSSSSHHTSSSTSSSSHSSSSSHSSHTTTSSSSSSTD
6xs6_A             SSSSSSHHHSHSSSSSSSTSSHHHTD                   TSSSSHHTSTSTSSSSTSSSSSHSSHTTTSSSTD
6xs6_B             SSSSSSHHHSHSSSSSSSTSSHHHTD                   TSSSSHHTSTHTHTTSTSSSSSHSSHTTTSSSTD
6xs6_C             SSSSSSHHHSHSSSSSSSTSSHHHTD                   TSSSSHHTSSHTSSTSHSSSSSHSSHTTTSSSTD
6xr8_A             SSSSSSHHHSHSSSSSSSTSSHTD             TTSHSSSHSSSSSHHTSSSTSSSSHSSSSSHSSHTTTSSSSSSTD
6xr8_B             SSSSSSHHHSHSSSSSSSTSSHTD             TTSHSSSHSSSSSHHTSSSTSSSSHSSSSSHSSHTTTSSSSSSTD
6xr8_C             SSSSSSHHHSHSSSSSSSTSSHTD             TTSHSSSHSSSSSHHTSSSTSSSSHSSSSSHSSHTTTSSSSSSTD
6xra_A
6xra_B
6xra_C
```

```
                  SD2
                  681      690       700       710       720       730       740       750       760
B.1.617.2         RRRARSVASQSIIAYTMSLGAENSVAYSNNSIAIPTNFTISVTTEILPVSMTKTSVDCTMYICGDSTECSNLLLQYGSFCTQLNR
SSSCPred200       THHHHHHHHHHHHHHHHHHHTSSHSHSSSSTSSSSSSSSSSSSSSSSSSSSSHHSSSSSSSSSSSSSHSHHHHHHHHHHTHHHHHHTH
SSSCPred100       HHHHHSSHHHHHHHHHHHHHTHHTSSSSSHSSHHSSHSSSSSSSSSSSSSSSSSSSSSHHSSSSSSSTSHHHHHHHHHHHTTHHHHHHH
SSSCPred          HHHSHHHSSSSSSSSSSSSSHHHHHHHHHHHTSSSSSSHHSSSSSSSSSSSSSSSSHHTSSSSSSSSSSSHHSHHHHHHHHHHHHHHHHHHH
B.1.617.1         RRRARSVASQSIIAYTMSLGAENSVAYSNNSIAIPTNFTISVTTEILPVSMTKTSVDCTMYICGDSTECSNLLLQYGSFCTQLNR
SSSCPred200       THHHHHHHHHHHHHHHHHHHTSSHSHSSSSTSSSSSSSSSSSSSSSSSSSSSHHSSSSSSSSSSSSSHSHHHHHHHHHHTHHHHHHTS
SSSCPred100       HHHHHSSHHHHHHHHHHHHHTHHTSSSSSHSSHHSSHSSSSSSSSSSSSSSSSSSSSSHHSSSSSSSTSHHHHHHHHHHHTTHHHHHHH
SSSCPred          HHHSHHHSSSSSSSSSSSSSHHHHHHHHHHHTSSSSSSHHSSSSSSSSSSSSSSSSHHTSSSSSSSSSSSHHSHHHHHHHHHHHHHHHHHHH
P.1               PRRARSVASQSIIAYTMSLGAENSVAYSNNSIAIPTNFTISVTTEILPVSMTKTSVDCTMYICGDSTECSNLLLQYGSFCTQLNR
SSSCPred200       HHHHHHHHHHHHHHHHHHHHTSSHSHSSSSTSSSSSSSSSSSSSSSSSSSSSHHSSSSSSSSSSSSSHSHHHHHHHHHHTHHHHHHTS
SSSCPred100       HHHHHHHHHHHHHHHHHHHHTHHTSSSSSHSSHHSSHSSSSSSSSSSSSSSSSSSSSSHHSSSSSSSTSHHHHHHHHHHHTTHHHHHHH
SSSCPred          HHTHHHHHHHSSSSSSSSSHHHHHHHHHHHTSSSSSSHHSSSSSSSSSSSSSSSSHHTSSSSSSSSSSSHHSHHHHHHHHHHHHHHHHHHH
B.1.351           PRRARSVASQSIIAYTMSLGVENSVAYSNNSIAIPTNFTISVTTEILPVSMTKTSVDCTMYICGDSTECSNLLLQYGSFCTQLNR
SSSCPred200       HHHHHHHHHHHHHHHHHHHHHHHHHSHSSSSTSSSSSSSSSSSSSSSSSSSSSHHSSSSSSSSSSSSSHSHHHHHHHHHHTHHHHHHTS
SSSCPred100       HHHHHHHHHHHHHHHHHHHHTHHHSSSSSHSSHSSSHSSSSSSSSSSSSSSSSSSSSSHHSSSSSSSTSHHHHHHHHHHHTTHHHHHHH
SSSCPred          HHTHHHHHHHHHHHSSSHHHHHHHHHHHHHHHSSSSSSHHSSSSSSSSSSSSSSSSHHTSSSSSSSSSSSHHSHHHHHHHHHHHHHHHHHHH
B.1.1.7           HRRARSVASQSIIAYTMSLGAENSVAYSNNSIAIPINFTISVTTEILPVSMTKTSVDCTMYICGDSTECSNLLLQYGSFCTQLNR
SSSCPred200       HHHHHHHHHHHHHHHHHHHHTSSHSSSSTSTSSSSSSSSSSSSSSSSSSSSSSHHSSSSSSSSSSSSSHSHHHHHHHHHHTHHHHHHTS
SSSCPred100       HHHHHSSHHHHHHHHHHHHHTHHTSSSSSHSSSSSSSSSSSSSSSSSSSSSSSSSSTSSSHHSSSSSSSTSHHHHHHHHHHHTTHHHHHHH
SSSCPred          HHSSHSSSSSSSSSSSSSSSHHHHHHHHHHHTSSSSSSSHSSSHSSSSSSSSSSSSHHTSSSSSSSSSSSHHSHHHHHHHHHHHHHHHHHHH
SARS-CoV-2   681  PRRARSVASQSIIAYTMSLGAENSVAYSNNSIAIPTNFTISVTTEILPVSMTKTSVDCTMYICGDSTECSNLLLQYGSFCTQLNR   765
SSSCPred200       HHHHHHHHHHHHHHHHHHHHTSSHSHSSSSTSSSSSSSSSSSSSSSSSSSSSHHSSSSSSSSSSSSSHSHHHHHHHHHHTHHHHHHTS
SSSCPred100       HHHHHHHHHHHHHHHHHHHHTHHTSSSSSHSSHHSSHSSSSSSSSSSSSSSSSSSSSSHHSSSSSSSTSHHHHHHHHHHHTTHHHHHHH
SSSCPred          HHTHHHHHHSSSSSSSSSHHHHHHHHHHHTSSSSSSHHSSSSSSSSSSSSSSSSHHTSSSSSSSSSSSHHSHHHHHHHHHHHHHHHHHHH
6vsb_A                  TSTTSSSSSSSSHTSSSSSSSSHHSSSSSHSSSSSSSSSSSSSSHSSSTSSSSHHHHHHHTTSHHHHHHHHHHHTHHHHHHHHH
6vsb_B                  TSTTSSSSSSSSHTSSSSSSSSHHSSSSSHSSSSSSSSSSSSSSHSSSTSSSSHHHHHHHTTSHHHHHHHHHHHTHHHHHHHHH
6vsb_C                  TSTTSSSSSSSSHTSSSSSTSSHTSSSSSHSSSSSSSSSSSSSSHSSSTSSSSHHHHHHHTTSHHHHHHHHHHHTHHHHHHHHH
6vxx_A                    TSSSSSSSSSHTSSSSSTSSHTSSSSSHSSSSSSSSSSSSSSHSSSTSSSSHHHHHHTSTSHHHHHHHHHHHTHHHHHHHHH
6vxx_B                    TSSSSSSSSSHTSSSSSTSSHTSSSSSHSSSSSSSSSSSSSSHSSSTSSSSHHHHHHTSTSHHHHHHHHHHHTHHHHHHHHH
6vxx_C                    TSSSSSSSSSHTSSSSSTSSHTSSSSSHSSSSSSSSSSSSSSHSSSTSSSSHHHHHHTSTSHHHHHHHHHHHTHHHHHHHHH
6vyb_A                     TSSSSSSSSHTSSSSSTSSHHSSSSSHSSSSSSSSSSSSSSHSSSTSSSSHHHHHHTSTSHHHHHHHHHHHTHHHHHHHHH
6vyb_B                    TSSSSSSSSSHTSSSSSTSSHHSSSSSHSSSSSSSSSSSSSSHSSSTSSSSHHHHHHTSTSHHHHHHHHHHHTHHHHHHHHH
6vyb_C                     TSSSSSSSSHTSSSSSTSSHHSSSSSHSSSSSSSSSSSSSSHSSSTSSSSHHHHHHTSTSHHHHHHHHHHHTHHHHHHHHH
6xs6_A                      TSSSSSSSHSSSSSSTSSHHSSSSSHSSSSSSSSSSSSSSHSSSSSSSSSHHHHHSSTSHHHHHHHHHHHTHHHHHHHHH
6xs6_B                      TSSSSSSSHSSSSSSTSSHHSSSSSHSSSSSSSSSSSSSSHSSSSSSSSSHHHHHSTTSHHHHHHHHHHHTHHHHHHHHH
6xs6_C                    TTSSSSSSSSHTSSSSSTSSHTSSSSSHSSSSSSSSSSSSSSHSSSSSSSSSHHHHHSSTSHHHHHHHHHHHTHHHHHHHHH
6xr8_A                    TSSSSSSSSSHTSSSSSHSSHHSSSSSHSSSSSSSSSSSSSSHSSSSSSSSSHHHHHHTSTSHHHHHHHHHHHTHHHHHHHHH
6xr8_B                    TSSSSSSSSSHTSSSSSTSSHHSSSSSHSSSSSSSSSSSSSSHSSSSSSSSSHHHHHHTSTSHHHHHHHHHHHTHHHHHHHHH
6xr8_C                    TSSSSSSSSSHTSSSSSTSSHHSSSSSHSSSSSSSSSSSSSSHSSSSSSSSTHHHHHHTSTSHHHHHHHHHHHTHHHHHHHHH
6xra_A                                  THHHHSSSSSSSSHSSSSSSSSSSSSSHSSSSTSSSHHHHHHHTHSHHHHHHHHHHHTTHHHHHHHH
6xra_B                                  THHHHSSSSSSSSHSSSSSSSSSSSSSHSSSSTSSSHHHHHHHTHSHHHHHHHHHHHTTHHHHHHHH
6xra_C                                  THHHHSSSSSSSSHSSSSSSSSSSSSSHSSSSTSSSHHHHHHHTHSHHHHHHHHHHHTTHHHHHHHH
```

```
                     UH                L                              FP                       CR
                         770       780       790       800       810       820       830       840       850
B.1.617.2                ALTGIAVEQDKNTQEVFAQVKQIYKTPPIKDFGGFNFSQILPDPSKPSKRSFIEDLLFNKVTLADAGFIKQYGDCLGDIAARDLI
SSSCPred200              HSHHSSSHHHHHHHHHHHHHHTSSSSSSSSHSSSSSSHHHSSSHHSHHTSSHHHHHHHHTSSSSSSHHHHHHHHHHHHHHHHHHHH
SSSCPred100              HHHHHTSHHSHHHHHHHHHHHHHHHHHSSSSHSSTTSSHHHHSSSHHSHTTSSHHHHHHHHHHSHHHHHHHHHHHHHHHHHHHHHHHH
SSSCPred                 HHHTSSSSSSHHHTHHHHHHHHHHHHHSSSSHHHSTSSHHHHSSSHHSSHHHHHHHHHHHHHHHSSSSHHHHHHHHHHHSHHHHHHHHHHH
B.1.617.1                ALTGIAVEQDKNTQEVFAQVKQIYKTPPIKDFGGFNFSQILPDPSKPSKRSFIEDLLFNKVTLADAGFIKQYGDCLGDIAARDLI
SSSCPred200              HSHHSSSHHHHHHHHHHHHHHTSSSSSSSSTSSSSSHHHHSSSHHSHHTSSHHHHHHHHTSSSSSSHHHHHHHHHHHHHHHHHHHH
SSSCPred100              HHHHHTSHHSHHHHHHHHHHHHHHHHHSSSSHTHTTSSHHHHSSSHHSHTTSSHHHHHHHHHHSHHHHHHHHHHHHHHHHHHHHHHHH
SSSCPred                 HHHTSSSSSSHHHTHHHHHHHHHHHHHSSSSHHHTTSSHHHHSSSHHSSHHHHHHHHHHHHHHHSSSSHHHHHHHHHHHSHHHHHHHHHHH
P.1                      ALTGIAVEQDKNTQEVFAQVKQIYKTPPIKDFGGFNFSQILPDPSKPSKRSFIEDLLFNKVTLADAGFIKQYGDCLGDIAARDLI
SSSCPred200              HSHHSSSHHHHHHHHHHHHHHTSSSSSSSSTSSSSSHHHHSSSHHSHHTSSHHHHHHHHTSSSSSSHHHHHHHHHHHHHHHHHHHH
SSSCPred100              HHHHHTSHHSHHHHHHHHHHHHHHHHHSSSSHTHTTSSHHHHSSSHHSHTTSSHHHHHHHHHHSHHHHHHHHHHHHHHHHHHHHHHHH
SSSCPred                 HHHTSSSSSSHHHTHHHHHHHHHHHHHSSSSHHHTTSSHHHHSSSHHSSHHHHHHHHHHHHHHHSSSSHHHHHHHHHHHSHHHHHHHHHHH
B.1.1.7                  ALTGIAVEQDKNTQEVFAQVKQIYKTPPIKDFGGFNFSQILPDPSKPSKRSFIEDLLFNKVTLADAGFIKQYGDCLGDIAARDLI
SSSCPred200              HSHHSSSHHHHHHHHHHHHHHTSSSSSSSSHSSSSSHHHHSSSHHSHHTSSHHHHHHHHTSSSSSSHHHHHHHHHHHHHHHHHHHH
SSSCPred100              HHHHHTSHHSHHHHHHHHHHHHHHHHHSSSSHSHTTSSHHHHSSSHHSHTTSSHHHHHHHHHHSHHHHHHHHHHHHHHHHHHHHHHHH
SSSCPred                 HHHTSSSSSSHHHHHHHHHHHHHHHHHSSSSHHHSTSSHHHHSSSHHSSHHHHHHHHHHHHHHHSSSSHHHHHHHHHHHSHHHHHHHHHHH
SARS-CoV-2     766       ALTGIAVEQDKNTQEVFAQVKQIYKTPPIKDFGGFNFSQILPDPSKPSKRSFIEDLLFNKVTLADAGFIKQYGDCLGDIAARDLI    850
SSSCPred200              HSHHSSSHHHHHHHHHHHHHHTSSSSSSSSTSSSSSHHHHSSSHHSHHTSSHHHHHHHHTSSSSSSHHHHHHHHHHHHHHHHHHHH
SSSCPred100              HHHHHTSHHSHHHHHHHHHHHHHHHHHSSSSHTHTTSSHHHHSSSHHSHTTSSHHHHHHHHHHSHHHHHHHHHHHHHHHHHHHHHHHH
SSSCPred                 HHHTSSSSSSHHHTHHHHHHHHHHHHHSSSSHHHTTSSHHHHSSSHHSSHHHHHHHHHHHHHHHSSSSHHHHHHHHHHHSHHHHHHHHHHH
6vsb_A                   HHHHHHHHHHHHHHHHHHTHSHSSSSSSHSSTTTTSSHHHHSSSHHTD  TSHHHHHHHHHSTTD
6vsb_B                   HHHHHHHHHHHHHHHHHHTHSHSSSSSSHSSTTTTSSHHHHSSSHHTD  TSHHHHHHHHHSTTD
6vsb_C                   HHHHHHHHHHHHHHHHHHTHSHSSSSSSHSSTTTTSSHHHHSSSHHTD  TSHHHHHHHHHSTTD
6vxx_A                   HHHHHHHHHHHHHHHHHHTHSHSSSSSSHSTTTTTSSHHHHSSSHHSHSTSSHHHHHHHHHHSTD
6vxx_B                   HHHHHHHHHHHHHHHHHHTHSHSSSSSSHSTTTTTSSHHHHSSSHHSHSTSSHHHHHHHHHHSTD
6vxx_C                   HHHHHHHHHHHHHHHHHHTHSHSSSSSSHSTTTTTSSHHHHSSSHHSHSTSSHHHHHHHHHHSTD
6vyb_A                   HHHHHHHHHHHHHHHHHHTHSHSSSSSSHSTTTTTSSHHHHSSSHHTDTTSSHHHHHHHHHHSTD
6vyb_B                   HHHHHHHHHHHHHHHHHHTHSHSSSSSSHSHSHTTSSHHHHSSSHHTDTTSSHHHHHHHHHHSTD
6vyb_C                   HHHHHHHHHHHHHHHHHHTHSHSSSSSSHSHTTTTSSHHHHSSSHHTDTTSSHHHHHHHHHHSTD
6xs6_A                   HHHHHHHHHHHHHHHHHHTHHTSSSSSSHSTTTTTSSHHHHSSSHHSHSSSSHHHHHHHHHHSTD
6xs6_B                   HHHHHHHHHHHHHHHHHHTHSHSSSSSSHSTTTTTSSTHHHSSSHHSHSSSSHHHHHHHHHHSTD
6xs6_C                   HHHHHHHHHHHHHHHHHHTHSHSSSSSSHSTTTTTSSTHHHSSSHHSHSSSSHHHHHHHHHHSTD
6xr8_A                   HHHHHHHHHHHHHHHHHHTHSHSSSSSSHSTTTTTSSHHHHSSSHHSHSTSSHHHHHHHHHHSSSHSSTTSSSHHHHSTHHHHHSHH
6xr8_B                   HHHHHHHHHHHHHHHHHHHHSHSSSSSSHSTTTTTSSHHHHSSTHHSHSTSSHHHHHHHHHHSSSHSSTTSSSHHHHSTHHHHHSHH
6xr8_C                   HHHHHHHHHHHHHHHHHHHHSTSSSSSSHSTTTTTSTHHHHSSTHHSHSTSSHHHHHHHHHHSSSHSSTTSSSHHHHSTHHHHHSHH
6xra_A                   HHHSTD
6xra_B                   HHHSTD
6xra_C                   HHHSTD
```

```
                    CR                                                            HR1
                    851      860       870       880       890       900       910       920       930
B.1.617.2           CAQKFNGLTVLPPLLTDEMIAQYTSALLAGTITSGWTFGAGAALQIPFAMQMAYRFNGIGVTQNVLYENQKLIANQFNSAIGKIQ
SSSCPred200         HHSHHSHSSSSSSHSSHHHHHHHHHHHHHHHHHHHHHHSSTSSSSSHHHHHHHHHHHTHTSSHHHHHHHHHHHHHHHHHHHHHHH
SSSCPred100         HSSSHSHSSSSSSHSSHHHHHHHHHHHHHHHHHHHSSSTHTSSSSSSTHHHHHHHHTTSSSSHHHHHHHHHHHHHHHHHHHHHHS
SSSCPred            HHHHHHTSSHSSSHSSHHHHHHHHHHHHHHHHHSTSSSSSTSSSSSSHSSTSSSSSHTSSSSHHHHHHHHHHHHHHHHHHHHHHH
B.1.617.1           CAQKFNGLTVLPPLLTDEMIAQYTSALLAGTITSGWTFGAGAALQIPFAMQMAYRFNGIGVTQNVLYENQKLIANQFNSAIGKIQ
SSSCPred200         HHSHHSHSSSSSSHSSHHHHHHHHHHHHHHHHHHHHHHSSTSSSSSHHHHHHHHHHHTHTSSHHHHHHHHHHHHHHHHHHHHHHH
SSSCPred100         HSSSHSHSSSSSSHSSHHHHHHHHHHHHHHHHHHHSSSTHTSSSSSSTHHHHHHHHTTSSSSHHHHHHHHHHHHHHHHHHHHHHS
SSSCPred            HHHHHHTSSHSSSHSSHHHHHHHHHHHHHHHHHSTSSSSSTSSSSSSHSSTSSSSSHSSSSSHHHHHHHHHHHHHHHHHHHHHHH
P.1                 CAQKFNGLTVLPPLLTDEMIAQYTSALLAGTITSGWTFGAGAALQIPFAMQMAYRFNGIGVTQNVLYENQKLIANQFNSAIGKIQ
SSSCPred200         HHSHHSHSSSSSSHSSHHHHHHHHHHHHHHHHHHHHHHSSTSSSSSHHHHHHHHHHHTHTSSHHHHHHHHHHHHHHHHHHHHHHH
SSSCPred100         HSSSHSHSSSSSSHSSHHHHHHHHHHHHHHHHHHHSSSTHTSSSSSSTHHHHHHHHTTSSSSHHHHHHHHHHHHHHHHHHHHHHS
SSSCPred            HHHHHHTSSHSSSHSSHHHHHHHHHHHHHHHHHSTSSSSSTSSSSSSHSSTSSSSSHSSSSSHHHHHHHHHHHHHHHHHHHHHHH
B.1.1.7             CAQKFNGLTVLPPLLTDEMIAQYTSALLAGTITSGWTFGAGAALQIPFAMQMAYRFNGIGVTQNVLYENQKLIANQFNSAIGKIQ
SSSCPred200         HHSHHSHSSSSSSHSSHHHHHHHHHHHHHHHHHHHHHHSSTSSSSSHHHHHHHHHHHTHTSSHHHHHHHHHHHHHHHHHHHHHHH
SSSCPred100         HSSSHSHSSSSSSHSSHHHHHHHHHHHHHHHHHHHSSSTHTSSSSSSTHHHHHHHHTTSSSSHHHHHHHHHHHHHHHHHHHHHHS
SSSCPred            HHHHHHTSSHSSSHSSHHHHHHHHHHHHHHHHHSTSSSSSTSSSSSSHSSTSSSSSHTSSSSHHHHHHHHHHHHHHHHHHHHHHH
SARS-CoV-2   851    CAQKFNGLTVLPPLLTDEMIAQYTSALLAGTITSGWTFGAGAALQIPFAMQMAYRFNGIGVTQNVLYENQKLIANQFNSAIGKIQ   935
SSSCPred200         HHSHHSHSSSSSSHSSHHHHHHHHHHHHHHHHHHHHHHSSTSSSSSHHHHHHHHHHHTHTSSHHHHHHHHHHHHHHHHHHHHHHH
SSSCPred100         HSSSHSHSSSSSSHSSHHHHHHHHHHHHHHHHHHHSSSTHTSSSSSSTHHHHHHHHTTSSSSHHHHHHHHHHHHHHHHHHHHHHS
SSSCPred            HHHHHHTSSHSSSHSSHHHHHHHHHHHHHHHHHSTSSSSSTSSSSSSHSSTSSSSSHSSSSSHHHHHHHHHHHHHHHHHHHHHHH
6vsb_A                TSHTTSSSSSSHSSHHHHHHHHHHHHHHHHHHTHHHHHSSSSSSHHHHHHHHHHHHHTSSHHHHHHHHHHHHHHHHHHHHHHH
6vsb_B                TSHSTSSSSSSHSSHHHHHHHHHHHHHHHHHHTHHHHHSSSSSSHHHHHHHHHHHHHTSSHHHHHHHHHHHHHHHHHHHHHHH
6vsb_C                TSHSTSSSSSSHSSHHHHHHHHHHHHHHHHHHTHHHHHSSSSSSHHHHHHHHHHHHHTSSHHHHHHHHHHHHHHHHHHHHHHH
6vxx_A                 THHTSSSSSSHSSHHHHHHHHHHHHHHHHHHTHHHHHTSSSSSHHHHHHHHHHHHHTSSHHHHHHHHHHHHHHHHHHHHHHH
6vxx_B                 THHTSSSSSSHSSHHHHHHHHHHHHHHHHHHTHHHHHTSSSSSHHHHHHHHHHHHHTSSHHHHHHHHHHHHHHHHHHHHHHH
6vxx_C                 THHTSSSSSSHSSHHHHHHHHHHHHHHHHHHTHHHHHTSSSSSHHHHHHHHHHHHHTSSHHHHHHHHHHHHHHHHHHHHHHH
6vyb_A                  THTSSSSSSHSSHHHHHHHHHHHHHHHHHHTHHHHHTSSSSSHHHHHHHHHHHHHTSSHHHHHHHHHHHHHHHHHHHHHHH
6vyb_B                 THHTSSSSSSHSSHHHHHHHHHHHHHHHHHHTHHHHHTSSSSSHHHHHHHHHHHHHTSSHHHHHHHHHHHHHHHHHHHHHHH
6vyb_C                   TTSSSSSSHSSHHHHHHHHHHHHHHHHHHTHHHHHTSSSSSHHHHHHHHHHHHHTSSHHHHHHHHHHHHHHHHHHHHHHH
6lxt_A                                                                           THHHHHHHHHHHHHHHHHHHHHHH
6xs6_A                 TTHTSSSSSSHSSHHHHHHHHHHHHHHHHHHTHHHHHHSSSSSHHHHHHHHHHHHHTSSHHHHHHHHHHHHHHHHHHHHHHH
6xs6_B                 TTHTSSSSSSHSSHHHHHHHHHHHHHHHHHHTHHHHHHSSSSSHHHHHHHHHHHHHTSSHHHHHHHHHHHHHHHHHHHHHHH
6xs6_C                 THHTSSSSSSHSSHHHHHHHHHHHHHHHHHHTHHHHHSSSSSSHHHHHHHHHHHHHTSSHHHHHHHHHHHHHHHHHHHHHHH
6xr8_A              HHHHHHTSSSSSSHSSHHHHHHHHHHHHHHHHHHTHHHHHSSSSSSHHHHHHHHHHHHHTSSHHHHHHTHHHHHHHHHHHHHHHH
6xr8_B              HHHHHHTSSSSSSHSSHHHHHHHHHHHHHHHHHHTHHHHHTSSSSSHHHHHHHHHHHHHTSSHHHHHHHHHHHHHHHHHHHHHHH
6xr8_C              HHHHHHTSSSSSSHSSHHHHHHHHHHHHHHHHHHTHHHHHSSSSSSHHHHHHHHHHHHHTSSHHHHHHHHHHHHHHHHHHHHHHH
6xra_A                                                                           THHHHHHHHHHHHHHHHHHHHHHH
6xra_B                                                                           THHHHHHHHHHHHHHHHHHHHHHH
6xra_C                                                                           THHHHHHHHHHHHHHHHHHHHHHH
```

		HR1 CH	
		940 950 960 970 980 990 1000 1010 1020	
B.1.617.2		DSLSSTASALGKLQNVVNQNAQALNTLVKQLSSNFGAISSVLNDILSRLDKVEAEVQIDRLITGRLQSLQTYVTQQLIRAAEIRA	
SSSCPred200		HHHHHHHHHHHHHHHHHHHHHHHHHHHHHHHHSHTTTHSHHHHHHHHHHHHHHHHHHHHHHHHHHHHHHHHHHHHHHHHHHHHHHH	
SSSCPred100		THHHHSSHHHHHHHHHHHHHHHHHHHHHHHHHSHHHHHHHHHHHHHHHHHHHHHHHHHHHHHHHHHHHHHHHHHHHHHHHHHHHH	
SSSCPred		HHHHHHHHHHHHHHHHHHHHHHHHHHHHHHHHHHHTHHHHHHHHHHHHHHHHHHHHHHHHHHHHHHHHHHHHHHHHHHHHHHHHH	
B.1.617.1		DSLSSTASALGKLQDVVNQNAQALNTLVKQLSSNFGAISSVLNDILSRLDKVEAEVQIDRLITGRLQSLQTYVTQQLIRAAEIRA	
SSSCPred200		HHHHHHHHHHHHHHHHHHHHHHHHHHHHHHHHSHTTSHSHHHHHHHHHHHHHHHHHHHHHHHHHHHHHHHHHHHHHHHHHHHHHHH	
SSSCPred100		THHHHHHHHHHHHHHHHHHHHHHHHHHHHHHHSHHHHHHHHHHHHHHHHHHHHHHHHHHHHHHHHHHHHHHHHHHHHHHHHHHHH	
SSSCPred		HHHHHHHHHHHHHHHHHHHHHHHHHHHHHHHHHHHTHHHHHHHHHHHHHHHHHHHHHHHHHHHHHHHHHHHHHHHHHHHHHHHHH	
P.1		DSLSSTASALGKLQDVVNQNAQALNTLVKQLSSNFGAISSVLNDILSRLDKVEAEVQIDRLITGRLQSLQTYVTQQLIRAAEIRA	
SSSCPred200		HHHHHHHHHHHHHHHHHHHHHHHHHHHHHHHHSTTTSHSHHHHHHHHHHHHHHHHHHHHHHHHHHHHHHHHHHHHHHHHHHHHHHH	
SSSCPred100		THHHHHHHHHHHHHHHHHHHHHHHHHHHHHHHSHHHHHHHHHHHHHHHHHHHHHHHHHHHHHHHHHHHHHHHHHHHHHHHHHHHH	
SSSCPred		HHHHHHHHHHHHHHHHHHHHHHHHHHHHHHHHHHHTHHHHHHHHHHHHHHHHHHHHHHHHHHHHHHHHHHHHHHHHHHHHHHHHH	
B.1.1.7		DSLSSTASALGKLQDVVNQNAQALNTLVKQLSSNFGAISSVLNDILARLDKVEAEVQIDRLITGRLQSLQTYVTQQLIRAAEIRA	
SSSCPred200		HHHHHHHHHHHHHHHHHHHHHHHHHHHHHHHHSSTTSHSHHHHHHHHHHHHHHHHHHHHHHHHHHHHHHHHHHHHHHHHHHHHHHH	
SSSCPred100		THHHHHHHHHHHHHHHHHHHHHHHHHHHHHHHSHHHHHHHHHHHHHHHHHHHHHHHHHHHHHHHHHHHHHHHHHHHHHHHHHHHH	
SSSCPred		HHHHHHHHHHHHHHHHHHHHHHHHHHHHHHHHHHHTHHHHHHHHHHHHHHHHHHHHHHHHHHHHHHHHHHHHHHHHHHHHHHHHH	
SARS-CoV-2	936	DSLSSTASALGKLQDVVNQNAQALNTLVKQLSSNFGAISSVLNDILSRLDKVEAEVQIDRLITGRLQSLQTYVTQQLIRAAEIRA	1020
SSSCPred200		HHHHHHHHHHHHHHHHHHHHHHHHHHHHHHHHSHTTSHSHHHHHHHHHHHHHHHHHHHHHHHHHHHHHHHHHHHHHHHHHHHHHHH	
SSSCPred100		THHHHHHHHHHHHHHHHHHHHHHHHHHHHHHHSHHHHHHHHHHHHHHHHHHHHHHHHHHHHHHHHHHHHHHHHHHHHHHHHHHHH	
SSSCPred		HHHHHHHHHHHHHHHHHHHHHHHHHHHHHHHHHHHTHHHHHHHHHHHHHHHHHHHHHHHHHHHHHHHHHHHHHHHHHHHHHHHHH	
6vsb_A		HHHHHSSTHTHHHHHHHHHHHHHHHHHHHHHHSHTTSHSHSHHHHHHHSSHHHHHHHHHHHHHHHHHHHHHHHHHHHHHHHHHHH	
6vsb_B		HHHHHSSTHTHHHHHHHHHHHHHHHHHHHHHHSHTTSHSHSHHHHHHHSSHHHHHHHHHHHHHHHHHHHHHHHHHHHHHHHHHHH	
6vsb_C		HHHHHSSTHTHHHHHHHHHHHHHHHHHHHHHHSHTTSHSHSHHHHHHHSSHHHHHHHHHHHHHHHHHHHHHHHHHHHHHHHHHHH	
6vxx_A		HHHHHSHHHHHHHHHHHHHHHHHHHHHHHHHHSHTTSHSHSHHHHHHHSSHHHHHHHHHHHHHHHHHHHHHHHHHHHHHHHHHHH	
6vxx_B		HHHHHSHHHHHHHHHHHHHHHHHHHHHHHHHHSHTTSHSHSHHHHHHHSSHHHHHHHHHHHHHHHHHHHHHHHHHHHHHHHHHHH	
6vxx_C		HHHHHSHHHHHHHHHHHHHHHHHHHHHHHHHHSHTTSHSHSHHHHHHHSSHHHHHHHHHHHHHHHHHHHHHHHHHHHHHHHHHHH	
6vyb_A		HHHHHSHHHHHHHHHHHHHHHHHHHHHHHHHHSHTTSHSHSHHHHHHHSSHHHHHHHHHHHHHHHHHHHHHHHHHHHHHHHHHHH	
6vyb_B		HHHHHSHHHHHHHHHHHHHHHHHHHHHHHHHHSHTTSHSHSHHHHHHHSSHHHHHHHHHHHHHHHHHHHHHHHHHHHHHHHHHHH	
6vyb_C		HHHHHSHHHHHHHHHHHHHHHHHHHHHHHHHHSHTTSHSHSHHHHHHHSSHHHHHHHHHHHHHHHHHHHHHHHHHHHHHHHHHHH	
6lxt_A		HHHHHHHHHHHHHHHHHHHHHHHHHHHHHHHHHHHHHHHHHHHHHHHHHHHHTD	
6xs6_A		HHHHHSHHHHHHHHHHHHHHHHHHHHHHHHHHSHTTSHSHSHHHHHHHSSHHHHHHHHHHHHHHHHHHHHHHHHHHHHHHHHHHH	
6xs6_B		HHHHHSHHHHHHHHHHHHHHHHHHHHHHHHHHSHTTSHSHSHHHHHHHSSHHHHHHHHHHHHHHHHHHHHHHHHHHHHHHHHHHH	
6xs6_C		HHHHHSHHHHHHHHHHHHHHHHHHHHHHHHHHSHTTSHSHSHHHHHHHSSHHHHHHHHHHHHHHHHHHHHHHHHHHHHHHHHHHH	
6xr8_A		HHHHHSHHHHHHHHHHHHHHHHHHHHHHHHHHSHTTSHSHSHHHHHHHSSHHHHHHHHHHHHHHHHHHHHHHHHHHHHHHHHHHH	
6xr8_B		HHHHHSHHHTHHHHHHHHHHHHHHHHHHHHHHSHTTSHSHSHHHHHHHSSHHHHHHHHHHHHHHHHHHHHHHHHHHHHHHHHHHH	
6xr8_C		HHHHHSHHHTHHHHHHHHHHHHHHHHHHHHHHSHTTSHSHSHHHHHHHSSHHHHHHHHHHHHHHHHHHHHHHHHHHHHHHHHHHH	
6xra_A		HHHHHHHHHHHHHHHHHHHHHHHHHHHHHHHHHHHHHHHHHHHHHHHHHHHHHHHHHHHHHHHHHHHHHHHHHHHHHHHHHHHHH	
6xra_B		HHHHHHHHHHHHHHHHHHHHHHHHHHHHHHHHHHHHHHHHHHHHHHHHHHHHHHHHHHHHHHHHHHHHHHHHHHHHHHHHHHHHH	
6xra_C		HHHHHHHHHHHHHHHHHHHHHHHHHHHHHHHHHHHHHHHHHHHHHHHHHHHHHHHHHHHHHHHHHHHHHHHHHHHHHHHHHHHHH	

		CH BH SD3	
		1021 1030 1040 1050 1060 1070 1080 1090 1100	
B.1.617.2		SANLAATKMSECVLGQSKRVDFCGKGYHLMSFPQSAPHGVVFLHVTYVPAQEKNFTTAPAICHDGKAHFPREGVFVSNGTHWFVT	
SSSCPred200		HHHHHHHHHHHHHHHSSSHSSTSTTSTSSHSSSSHSSSSSSSSHHSSSSSSSSSTSSSSSSSSTSTTSSSSSSSSSSSSSSTSSSSSS	
SSSCPred100		HHHHHHHHHHHHHHHTSSHSSSSTTSTSSHSSSHHSSSSHSSSSSSSSSSSHHSSSSSHSSSSSSHHHHHSHSSTSSSHHTSSSSSS	
SSSCPred		HHHHHHHHHHHHHHHSSSHSSTSTTSTSSHSSSSSHSSTTSSSSSSSSSSSSSSSSSSSSSSSSSSSSTTSSSSSSSTSSSSHTSSSSSS	
B.1.617.1		SANLAATKMSECVLGQSKRVDFCGKGYHLMSFPQSAPHGVVFLHVTYVPAHEKNFTTAPAICHDGKAHFPREGVFVSNGTDWFVT	
SSSCPred200		HHHHHHHHHHHHHHHSSSHSSTSTTSTSSHSSSSHSSSSSSSSHSSSSSSSSSHTSHSSSSSTSTTSSSSSSSSSSSSSSTHHSSHS	
SSSCPred100		HHHHHHHHHHHHHHHTSSHSSSSTTSTSSHSSSHHSSSSHSSSSSSSSSSSHHHSSSSSSSSSSSSHHHHHSHSHSSSSHHTSSSSSS	
SSSCPred		HHHHHHHHHHHHHHHSSSHSSSSTTSTSSHSSSSSSSSSTTSSSSSSSSSSSHHSHSSSSSSSSSSSTTSSSSSSSTSSSSHTHSSSSH	
P.1		SANLAAIKMSECVLGQSKRVDFCGKGYHLMSFPQSAPHGVVFLHVTYVPAQEKNFTTAPAICHDGKAHFPREGVFVSNGTHWFVT	
SSSCPred200		HHHHHHHHHHHHHHHTSSHSSTSTTSTSSHSSSSHSSSSSSSSHHSSSSSSSSSTSSSSSSSSTSTTSSSSSSSSSSSSSSTSSSSSS	
SSSCPred100		HSTHHHHHHHHHHHHTSSHSSSSTTSTSSHSSSHHSSSSHSSSSSSSSSSSHHSSSSSHSSSSSSHHHHHSHSSTSSSHHTSSSSSS	
SSSCPred		HHHHHHHHHHHHHHHSSSHSSSSTTSTSSHSSSSSSSSSTTSSSSSSSSSSSSSSSSSSSSSSSSSSSTTSSSSSSSTSSSSTTSSSSSS	
B.1.1.7		SANLAATKMSECVLGQSKRVDFCGKGYHLMSFPQSAPHGVVFLHVTYVPAQEKNFTTAPAICHDGKAHFPREGVFVSNGTHWFVT	
SSSCPred200		HHHHHHHHHHHHHHHSSSHSSTSTTSTSSHSSSSHSSSSSSSSHHSSSSSSSSSTSSSSSSSSTSTTSSSSSSSSSSSSSSTSSSSSS	
SSSCPred100		HHHHHHHHHHHHHHHTSSHSSSSTTSTSSHSSSHHSSSSHSSSSSSSSSSSHHSSSSSHSSSSSSHHHHHSHSSTSSSHHTSSSSSS	
SSSCPred		HHHHHHHHHHHHHHHSSSHSSSSTTSTSSHSSSSSSSSSTTSSSSSSSSSSSSSSSSSSSSSSSSSSSTTSSSSSSSTSSSSHTSSSSSS	
SARS-CoV-2	1021	SANLAATKMSECVLGQSKRVDFCGKGYHLMSFPQSAPHGVVFLHVTYVPAQEKNFTTAPAICHDGKAHFPREGVFVSNGTHWFVT	1105
SSSCPred200		HHHHHHHHHHHHHHHSSSHSSTSTTSTSSHSSSSHSSSSSSSSHHSSSSSSSSSTSSSSSSSSTSTTSSSSSSSSSSSSSSTSSSSSS	
SSSCPred100		HHHHHHHHHHHHHHHTSSHSSSSTTSTSSHSSSHHSSSSHSSSSSSSSSSSHHSSSSSHSSSSSSHHHHHSHSSTSSSHHTSSSSSS	
SSSCPred		HHHHHHHHHHHHHHHSSSHSSSSTTSTSSHSSSSSSSSSTTSSSSSSSSSSSSSSSSSSSSSSSSSSSTTSSSSSSSTSSSSTTSSSSSS	
6vsb_A		HHHHHHHHHHHHHHHSSSHSSTHTTSTSSHSSSSSSSHHSSSSSSSSSSSSSTSSSSSSSHSSSSTTSSSSSHSTSSSSTTHSSSSS	
6vsb_B		HHHHHHHHHHHHHHHSSSHSSTHTTSTSSHSSSSSSSHHSSSSSSSSSSSSSTSSSSSSSHSSSSTTSSTSSHSTSSSSTTHSSSSS	
6vsb_C		HHHHHHHHHHHHHHHSSSHSSTHTTSTSSHSSSSSSSHTSSSSSSSSSSSSSTSSSSSSSHSSSSTTSSSSSHTTSSSSHTHSSSSS	
6vxx_A		HHHHHHHHHHHHHHHSSSHSSTHTTSTSSHSSSSSSSSTSSSSSSSSSSSSHSSSSSSSSHSSSHHTSSSSSHSTSSSSHTHSSSSS	
6vxx_B		HHHHHHHHHHHHHHHSSSHSSTHTTSTSSHSSSSSSSSTSSSSSSSSSSSSHSSSSSSSSHSSSHHTSSSSSHSTSSSSHTHSSSSS	
6vxx_C		HHHHHHHHHHHHHHHSSSHSSTHTTSTSSHSSSSSSSSTSSSSSSSSSSSSHSSSSSSSSHSSSHHTSSSSSHSTSSSSHTHSSSSS	
6vyb_A		HHHHHHHHHHHHHHHSSSHSSTHTTSTSSHSSSSSSSSTSSSSSSSSSSSSHSSSSSSSSHSSSHHTSSSSSHSTSSSSHTHSSSSS	
6vyb_B		HHHHHHHHHHHHHHHSSSHSSTHTTSTSSHSSSSSSSSTSSSSSSSSSSSSHSSSSSSSSHSSSHHTSSSSSHSTSSSSHTHSSSSS	
6vyb_C		HHHHHHHHHHHHHHHSSSHSSTHTTSTSSHSSSSSSSSTSSSSSSSSSSSSHSSSSSSSSHSSSHHTSSSSSHTTSSSSHTHSSSSS	
6xs6_A		HHHHHHHHHHHHHHHSSSHSSTHTTSTSSHSSSSSSSSTSSSSTSSSSSSHSSSSSSSSHSSSHTTSSSSSHSTSSSSHTHSSSSS	
6xs6_B		HHHHHHHHHHHHHHHSSSHSSTHTTSTSSHSSSSSSSSTSSSSTSSSSSSSTSSSSSSSHSSSHTTSSSSSHSTSSSSHTHSSSSS	
6xs6_C		HHHHHHHHHHHHHHHSSSHSSTHTTSTSSHSSSSSSSSTSSSSTSSSSSSHSSSSSSSSHSSSHTTSSSSSHSTSSSSHTHSSSSS	
6xr8_A		HHHHHHHHHHHHHHHSSSHSSTHTTSTSSHSSSSSSSSTSSSSSSSSSSSSHTSSSSSSSHSSSHTTSSSSSHSTSSSSHTHSSSSS	
6xr8_B		HHHHHHHHHHHHHHHSSSHSSTHTTSTSSHSSSSSSSSTSSSSSSSSSSSSHTSSSSSSSHSSSHHTSSSSSHSTSSSSHTHSSSSS	
6xr8_C		HHHHHHHHHHHHHHHSSSHSSTHTTSTSSHSSSSSSSSTSSSSSSSSSSSSHTSSSSSSSHSSSHTTSSSSSHSTSSSSHTHSSSSS	
6xra_A		HHHHHHHHHHHHHHHSSSHSSTSTTSTSSHSSSSSSSHTSSSSSSSSSSSSHSSSSSSSSHSSSSTTSSSSSSTSSTSSSHHSSSSS	
6xra_B		HHHHHHHHHHHHHHHSSSHSSTSTTSTSSHSSSSSSSHTSSSSSSSSSSSSHSSSSSSSSHSSSSTTSSSSSSTSSTSSSHHSSSSS	
6xra_C		HHHHHHHHHHHHHHHSSSHSSTSTTSTSSHSSSSSSSHTSSSSSSSSSSSSHSSSSSSSSHSSSSTTSSSSSSTSSTSSSHHSSSSS	

```
                          SD3                                                     HR2
                              1110      1120      1130      1140      1150      1160      1170      1180      1190
B.1.617.2                 QRNFYEPQIITTDNTFVSGNCDVVIGIVNNTVYDPLQPELDSFKEELDKYFKNHTSPDVDLGDISGINASVVNIQKEIDRLNEVA
SSSCPred200               HHSHHSSSSSSHHHSSSTTSSHSSSSSSSSSHHHSHSSSSHHHHHHHHHHHHHSSHSSHHSSHTTSHSSSSSSSSSHHHHHHHHHHHH
SSSCPred100               SHSHSSSSSSSSSHHSHHHTSSHSSSSSSSSSHHHHHSSHHHHHHHHHHHHHHHHHHSSSSSSSHSSSHSSSSSSSSSHHHHHHHHHHHH
SSSCPred                  HSSSSSSSSSSSSTHHSSTHSHSSSSSSSHSSSSSSSTHHHHHHHHHHHHHHSHHHSSHTSSHTSHHHSSSSSSSSHHHHHHHHHHHH
B.1.617.1                 QRNFYEPQIITTDNTFVSGNCDVVIGIVNNTVYDPLQPELDSFKEELDKYFKNHTSPDVDLGDISGINASVVNIQKEIDRLNEVA
SSSCPred200               HHSHHSSSSSSHHHSSSHTSSHSSSSSSSSSHHHSHSSSSHHHHHHHHHHHHHSSHSSHHSSHTTSHSSSSSSSSSHHHHHHHHHHHH
SSSCPred100               SHSTSSSSSSSSSHHSHHHTSSHSSSSSSSSSHHHHHSSHHHHHHHHHHHHHHHHHHSSSSSSSHSSSHSSSSSSSSSHHHHHHHHHHHH
SSSCPred                  HSSHSSSSSSSSSTHHSSTHSHSSSSSSSHSSSSSSSTHHHHHHHHHHHHHHSHHHSSHTSSHTSHHHSSSSSSSSHHHHHHHHHHHH
P.1                       QRNFYEPQIITTDNTFVSGNCDVVIGIVNNTVYDPLQPELDSFKEELDKYFKNHTSPDVDLGDISGINASFVNIQKEIDRLNEVA
SSSCPred200               HHSHHSSSSSSHHHSSSTTSSHSSSSSSSSSHHHSHSSSSHHHHHHHHHHHHHSSHSSHHSSHTTSSTSSHSHHHHHHHHHHHHHHH
SSSCPred100               SHSHSSSSSSSSSHHSHHHTSSHSSSSSSSSSHHHHHSSHHHHHHHHHHHHHHHHHHSSSSSSSHSSSHSSSHHHHHHHHHHHHHHHH
SSSCPred                  HSSSSSSSSSSSSTHHSSTHSHSSSSSSSHSSSSSSSTHHHHHHHHHHHHHHHHHHHSSHTSSHTSHHHHHSSSSSHHHHHHHHHHHH
B.1.1.7                   QRNFYEPQIITTHNTFVSGNCDVVIGIVNNTVYDPLQPELDSFKEELDKYFKNHTSPDVDLGDISGINASVVNIQKEIDRLNEVA
SSSCPred200               HHSSHSSSSSSHSHHHHHTSSHSSSSSSSSSHHHSHSSSSHHHHHHHHHHHHHSSHSSHHSSHTTSHSSSSSSSSSHHHHHHHHHHHH
SSSCPred100               SHSHSSSSSSSSSHHHHHHTTSHSSSSSSSSSHHHHHSSHHHHHHHHHHHHHHHHHHSSSSSSSHSSSHSSSSSSSSSHHHHHHHHHHHH
SSSCPred                  HSSSSSSSSSSSSHHHHHTHSHSSSSSSSHSSSSSSSTHHHHHHHHHHHHHHSHHHSSHTSSHTSHHHSSSSSSSSHHHHHHHHHHHH
SARS-CoV-2   1106         QRNFYEPQIITTDNTFVSGNCDVVIGIVNNTVYDPLQPELDSFKEELDKYFKNHTSPDVDLGDISGINASVVNIQKEIDRLNEVA    1190
SSSCPred200               HHSHHSSSSSSHHHSSSTTSSHSSSSSSSSSHHHSHSSSSHHHHHHHHHHHHHSSHSSHHSSHTTSHSSSSSSSSSHHHHHHHHHHHH
SSSCPred100               SHSHSSSSSSSSSHHSHHHTSSHSSSSSSSSSHHHHHSSHHHHHHHHHHHHHHHHHHSSSSSSSHSSSHSSSSSSSSSHHHHHHHHHHHH
SSSCPred                  HSSSSSSSSSSSSTHHSSTHSHSSSSSSSHSSSSSSSTHHHHHHHHHHHHHHSHHHSSHTSSHTSHHHSSSSSSSSHHHHHHHHHHHH
6vsb_A                    SHHSSSSSSSSSHHHSSSSTSHHHSSTSSSSSSSSSHHHHHHTD
6vsb_B                    SHHSSSSSSSSSHHHSSSSTSTHHSSTSSSSSSSSSHHHHHHTD
6vsb_C                    SHHSSTSSSSSHHHSSSSTSHHHSSTSSSSSSSSSHHHHHHTD
6vxx_A                    SHHSSTSSSSSHHHSSSSTSHHHSSTSSSSSSSSSHHHHHHHTD
6vxx_B                    SHHSSTSSSSSHHHSSSSTSHHHSSTSSSSSSSSSHHHHHHHTD
6vxx_C                    SHHSSTSSSSSHHHSSSSTSHHHSSTSSSSSSSSSHHHHHHHTD
6vyb_A                    SHHSSTSSSSSHHHSSSSTSHHHSSTSSSSSSSSSHHHHHHHTD
6vyb_B                    SHHSSTSSSSSHHHSSSSTSHHHSSTSSSSSSSSSHHHHHHHTD
6vyb_C                    SHHSSTSSSSSHHHSSSSTSHHHSSTSSSSSSSSSHHHHHHHTD
6lxt_A                                                                              TSSSSHHHSSSSSSSHHHHHHHHHHHH
6lvn_A                                                                                   THHHHHHHHHHHHHHHHHHHHH
6xs6_A                    SHHSSSSSSSSSHHHSSSSTSHHHSSTSSSSSTD
6xs6_B                    SHHSSSSSSSSSHHHSSSSTSHHHSSTSSSSSTD
6xs6_C                    SHHSSSSSSSSSHHHSSSTTSHHHSSTSSTSSTD
6xr8_A                    SHHSSTSSSSSHHHSSSSTSHHHSSTSSSSSSSSSHHHHHHHHHHHHHHHHHHHHHHHHTD
6xr8_B                    SHHSSTSSSSSHHHSSSSTSHHHSSTSSSSSSSSSHHHHHHHHHHHHHHHHHHHHHHHHTD
6xr8_C                    SHHSSTSSSSSHHHSSSSTSHHHSSTSSSSSSSSSHHHHHHHHHHHHHHHHHHHHHHHHTD
6xra_A                    SHHSSTSSSSSHHHSSSSSTTSSSSSSSSSTSSSSSSSTSSSSHHHHHHHHHSSTSSSSSSSSSTHHHHTD     THHHHHHHHHH
6xra_B                    SHHSSTSSSSSHHHSSSSSTTSSSSSSSSSTSSSSSSSTSSSSHHHHHHHHHSSTSSSSSSSSSTHHHHTD     THHHHHHHHHH
6xra_C                    SHHSSTSSSSSHHHSSSSSTTSSSSSSSSSTSSSSSSSTSSSSHHHHHHHHHSSTSSSSSSSSSTHHHHTD     THHHHHHHHHH
```

		HR2 TM		
		1191 1200 1210 1220 1230 1240 1250 1260 1270		
B.1.617.2		KNLNESLIDLQELGKYEQYIKWPWYIWLGFIAGLIAIVMVTIMLCCMTSCCSCLKGCCSCGSCCKFDEDDSEPVLKGVKLHYT		
SSSCPred200		HHHHHHHHHHHHHHHHHHHHHHHHHHHHHHHHHHHHHHHHHHHHHHHHTSHHHSSTHSHSTTHSSTSSSHSSSHSSTSSSSSS		
SSSCPred100		HHHHHHTSSHSSSHHHHHHSHSHHHHHHHHHHHHHHHHHHHHHHHHHHHSSHHHSTSHHHTSSSSSHHHHHSSSSSTSSSSSS	0.718332	
SSSCPred		HHHHHHHSSHHHHHHHHHHHSSSHHHHHHHHHHHHHHHHHHHHHHHHHHHHHHHHTTSHHHTHHSSSSHHSHSHHSSTSSSSSS	0.707317	0.713611
B.1.617.1		KNLNESLIDLQELGKYEQYIKWPWYIWLGFIAGLIAIVMVTIMLCCMTSCCSCLKGCCSCGSCCKFDEDDSEPVLKGVKLHYT		
SSSCPred200		HHHHHHHHHHHHHHHHHHHHHHHHHHHHHHHHHHHHHHHHHHHHHHHHTSHHHSSTHSHSTTHSSTSTSHSSSHSSTSSSSSS		
SSSCPred100		HHHHHHTSSHSSSHHHHHHSHSHHHHHHHHHHHHHHHHHHHHHHHHHHHSSHHHSTSHHHTSSSSSHHHHHSSSSSTSSSSSS	0.728987	
SSSCPred		HHHHHHHSSHHHHHHHHHHHSSSSHHHHHHHHHHHHHHHHHHHHHHHHHHHHHHHTTSHHHTHHSSSSHHSHSHHSSTSSSSSS	0.710919	0.717203
P.1		KNLNESLIDLQELGKYEQYIKWPWYIWLGFIAGLIAIVMVTIMLCCMTSCCSCLKGCCSCGSCCKFDEDDSEPVLKGVKLHYT		
SSSCPred200		HHHHHHHHHHHHHHHHHHHHHHHHHHHHHHHHHHHHHHHHHHHHHHHHTSHHHSSTHSHSTTHSSTSSSHSSSHSSTSSSSSS		
SSSCPred100		HHHHHHTSSHSSSHHHHHHSHSHHHHHHHHHHHHHHHHHHHHHHHHHHHSSHHHSTSHHHTSSSSSHHHHHSSSSSTSSSSSS	0.714061	
SSSCPred		HHHHHHHSSHHHHHHHHHHHSSSSHHHHHHHHHHHHHHHHHHHHHHHHHHHHHHHTTSHHHTHHSSSSHHSHSHHSSTSSSSSS	0.706991	0.710919
B.1.1.7		KNLNESLIDLQELGKYEQYIKWPWYIWLGFIAGLIAIVMVTIMLCCMTSCCSCLKGCCSCGSCCKFDEDDSEPVLKGVKLHYT		
SSSCPred200		HHHHHHHHHHHHHHHHHHHHHHHHHHHHHHHHHHHHHHHHHHHHHHHHTSHHHSSTHSHSTTHSSTSSSHSSSHSSTSSSSSS		
SSSCPred100		HHHHHHSSSHSSSHHHHHHSHSHHHHHHHHHHHHHHHHHHHHHHHHHHHSSHHHSTSHHHTSSSSSHHHHHSSSSSTSSSSSS	0.723622	
SSSCPred		HHHHHHHSSHHHHHHHHHHHSSSHHHHHHHHHHHHHHHHHHHHHHHHHHHHHHHHTTSHHHTHHSSSSHHSHSHHSSTSSSSSS	0.709449	0.711024
SARS-CoV-2	1191	KNLNESLIDLQELGKYEQYIKWPWYIWLGFIAGLIAIVMVTIMLCCMTSCCSCLKGCCSCGSCCKFDEDDSEPVLKGVKLHYT	1273	
SSSCPred200		HHHHHHHHHHHHHHHHHHHHHHHHHHHHHHHHHHHHHHHHHHHHHHHHTSHHHSSTHSHSTTHSSTSSSHSSSHSSTSSSSSS		
SSSCPred100		HHHHHHTSSHSSSHHHHHHSHSHHHHHHHHHHHHHHHHHHHHHHHHHHHSSHHHSTSHHHTSSSSSHHHHHSSSSSTSSSSSS	0.717989	
SSSCPred		HHHHHHHSSHHHHHHHHHHHSSSSHHHHHHHHHHHHHHHHHHHHHHHHHHHHHHHTTSHHHTHHSSSSHHSHSHHSSTSSSSSS	0.705420	0.717203
6lxt_A		HHHHHHSSSSSTD		
6lvn_A		HHHHHHHHHHHTD		
6xs6_A				
6xs6_B				
6xs6_C				
6xr8_A				
6xr8_B				
6xr8_C				
6xra_A		HHHHHHTD		
6xra_B		HHHHHHTD		
6xra_C		HHHHHHTD		
